# CRLF3 plays a key role in the final stage of platelet genesis and is a potential therapeutic target for thrombocythaemia

**DOI:** 10.1182/blood.2021013113

**Published:** 2022-04-07

**Authors:** Cavan Bennett, Moyra Lawrence, Jose A. Guerrero, Simon Stritt, Amie K. Waller, Yahui Yan, Richard W. Mifsud, Jose Ballester-Beltran, Ayesha Baig, Annett Mueller, Louisa Mayer, James Warland, Christopher J. Penkett, Parsa Akbari, Thomas Moreau, Amanda L. Evans, Souradip Mookerjee, Gary J. Hoffman, Kourosh Saeb-Parsy, David J. Adams, Amber L. Couzens, Markus Bender, Wendy N. Erber, Bernhard Nieswandt, Randy J. Read, Cedric Ghevaert

**Affiliations:** 1 Department of Haematology, University of Cambridge and NHS Blood and Transplant, Cambridge Blood Centre, Long Road, Cambridge CB2 0PT, UK; 2 Cambridge Stem Cell Institute, University of Cambridge, Jeffrey Cheah Biomedical Centre, Puddicombe Way, Cambridge CB2 0AW, UK; 3 Institute of Experimental Biomedicine, University Hospital and University of Würzburg, Josef-Schneider-Str. 2, 97080 Würzburg, Germany; 4 Cambridge Institute for Medical Research and Department of Haematology, University of Cambridge, Wellcome Trust/MRC Building, Hills Road, Cambridge CB2 0XY, England; 5 MRC/BHF Cardiovascular Epidemiology Unit, Department of Public Health and Primary Care, University of Cambridge, Strangeways Research Laboratory, Wort’s Causeway, Cambridge CB1 8RN, UK; 6 Department of Human Genetics, The Wellcome Trust Sanger Institute, Wellcome Trust Genome Campus, Hinxton, Cambridge CB10 1HH, UK; 7 Medical School, Faculty of Health and Medical Sciences, The University of Western Australia, Crawley, WA, 6099, Australia; 8 Department of Surgery, University of Cambridge, and NIHR Cambridge Biomedical Research Centre, Cambridge, UK; 9 The Wellcome Trust Sanger Institute, Wellcome Genome Campus, Cambridge, CB10 1HH, UK; 10 Lunenfeld-Tanenbaum Research Institute, Sinai Health System, Toronto, Ontario, M5G 1X5, Canada

## Abstract

The process of platelet production has so far been understood to be a two-stage process: megakaryocyte (MK) maturation from haematopoietic stem cells followed by proplatelet formation, with each phase regulating the peripheral blood platelet count. Proplatelet formation releases “beads-on-a-string” preplatelets into the blood stream that undergo fission into mature platelets. For the first time, we show that preplatelet maturation is a third, tightly regulated, critical process akin to cytokinesis that regulates platelet count. We show that deficiency in cytokine receptor-like factor 3 (CRLF3) in mice leads to an isolated and sustained 25-48% reduction in the platelet count without any effect on other blood cell lineages. We show that *Crlf3^-/-^* preplatelets have increased microtubule stability, possibly due to increased microtubule glutamylation via CRLF3’s interaction with key members of the Hippo pathway. Using a mouse model of JAK2V617F Essential Thrombocythaemia (ET), we show that a lack of CRLF3 leads to a long-term lineage-specific normalisation of the platelet count. We thereby postulate that targeting CRLF3 has therapeutic potential for treatment of thrombocythaemia.

## Introduction

Platelets are small (2-4μm) anucleated blood cells, whose main function is to form thrombi upon vessel injury. Thrombi can form inappropriately on atherothrombotic plaques causing heart attacks or strokes. Platelets are produced by megakaryocytes (MKs), which derive from haematopoietic stem cells (HSCs). To release platelets, MKs produce long cytoskeletal processes (proplatelets), which extend into the circulation where large fragments (preplatelets) are shed^1,2^. Preplatelets undergo fission to form mature discoid platelets^3^.

Thrombocytopenia (platelet count <150x10^9^/L) can be caused by lack of platelet production or peripheral consumption of platelets. Multiple genetic disorders affect platelet production, which can be broadly separated into two groups: disorders that affect MK differentiation (the “first stage” of platelet production) and disorders that affect proplatelet formation (the “second stage”). Unsurprisingly, mutations associated with the latter are often in genes associated with the actin-tubulin cytoskeleton, such as *TUBB1*^4^, *MHY9*^5^, *FLNA*^6^, *ACTN1*^7^, *TPM4*^8^ and *DIAPH1*^9^.

Thrombocythaemia (platelet count >450x10^9^/L) due to acquired clonal mutations in HSCs is termed Essential Thrombocythaemia (ET). The major mutations seen in ET affect the tyrosine kinase, Janus Kinase 2 (JAK2)^10–13^, the endoplasmic reticulum chaperone, Calreticulin^14,15^, and the thrombopoietin (TPO) receptor, MPL^16,17^. ET patients typically have high survival rates and the main complications are serious thrombotic events (affecting 1/3 of patients). The therapeutic management in ET patients is primarily to prevent thrombotic events^18^ with agents that reduce platelet function (low dose aspirin) and cytoreductive agents that reduce MK production (hydroxyurea and anagrelide).

Cytokine Receptor Like Factor 3 (CRLF3) is a poorly studied but widely expressed 488 amino acid protein encoded in chromosome 17q11.2 in a region that is deleted in Neurofibromatosis type 1. Overexpression of CRLF3 in cell lines implicated it in cell-cycle progression^19^.

We show for the first time that preplatelet fission to platelets is a critical rate-limiting step of platelet production (the “third stage”) and that CRLF3 plays a central role in this process by controlling microtubule stability, potentially through its interaction with Hippo pathway proteins. We also show that CRLF3 deficiency leads to an isolated and sustained correction of platelet count in a mouse model of ET, showing its potential as a novel therapeutic target for ET.

## Methods

### Ethics

This research was regulated by the Animals (Scientific Procedures) Act 1986 Amendment Regulations 2012, UK (Project Licence 70/8406) and the district government of Lower Franconia (Bezirksregierung Unterfranken). Human blood samples were obtained from healthy volunteers under local ethics approval (HBREC.2018.13). Ethical approval for WGS was provided by the East of England Cambridge South national research ethics committee 13/EE/0325. Informed consent was received as per the Declaration of Helsinki.

### Animals

Generation of *Crlf3^-/-^* (*Crlf3^tm1b(KOMP)Wtsi^*) mice was performed as previously reported^20–22^. Mice were maintained on C57Bl/6 background. Age, sex matched control animals were used in all experiments.

### Complete Blood Counts

ETDA anticoagulated whole blood taken from the tail vein or inferior vena cava was run on a ABC blood counter (Woodley) or Vet abc Plus+ (Scil).

### *In vitro* platelet assays

Platelet response to agonists^23^, platelet spreading^24^, expression of major platelet receptors^25^, platelet survival^26^ and cold induced microtubule disassembly^9^ were performed as previously described. Thrombus formation assays was performed with heparin anticoagulated whole blood flowed into Vena8 Fluoro+ Biochips (Cellix) pre-coated with HORM Collagen (Takeda) as described^27^. Five images per channel were obtained using an EVOS fl microscope and AMG camera and analysed in ImageJ.

### Platelet depletion

Mice were injected intraperitoneally with 0.6μg/g anti-CD42b (Emfret Analytics) in PBS. Mice were bled for CBC from the tail vein at 0, 24, 48, 72 and 96 hours post injection into EDTA-coated tubes (Microvette).

### Splenectomy

Platelet and preplatelet counts were determined pre-/post-splenectomy by flow cytometry as described^28^. Preplatelets were counted as GPV^+^/GPIIbIIIa^+^ events that have larger forward/sideward scatter characteristic than platelets. Heparinised blood incubated with antibodies against activated GPIIbIIIa (M023-2) and CD62P (M130-1; both Emfret Analytics) eliminated preplatelets as microaggregates (Supplemental Figure 4D).

### Platelet imaging

Transmission and scanning electron microscopy, Rapid Romanowsky staining and confocal microscopy were performed as detailed in the Supplemental Material. Two-photon intravital microscopy was performed as described^29^.

### MK cultures

Mouse bone marrow cells were prepared, MKs cultured and mature MKs purified as described^23,30^. Differentiation and ploidy of cultured MKs were analysed as described^23^ using propidium iodide (Sigma-Aldrich). Samples were acquired using a Beckman Coulter Cyan flow cytometer and analysed using Kaluza Analysis version 1.5a software (Beckman Coulter). Details of *in vitro* proplatelet formation are in the Supplemental Material.

### iPSC-MKs

Forward programming of TAP-tagged (see Supplemental Material) and untagged iPSCs to iPSC-MKs was performed as previously described^27^. Proplatelet formation was carried out as above. For protein distribution, iPSC-MKs attached to coverslips were fixed with 10% neutral buffered formalin (Sigma-Aldrich) and stained with antibodies against α-tubulin (T5168), FLAG (F1804; both Sigma-Aldrich) and DAPI. Images were acquired using a Leica Sp5 inverted confocal microscope with the 63x immersion-oil objective and the Leica LAS 2.1 software and analysed using ImageJ.

### Structural solution CRLF3

Purified murine CRLF3 protein (amino acid 174-442) was obtained using standard cloning, production, and purification methods. Crystallisation was screened by the vapour diffusion method in 96-well sitting drop plates set up with a Nanodrop Screenmaker 96+8 (Innovadyne Technologies). Diffraction data were recorded at Diamond Light Source (Didcot, UK). The structure was determined by Hg-SAD using the AutoSol-wizard^31^ of the PHENIX suite^32^ with a dataset collected with a wavelength of 1.006 Å from a crystal grown in 20% PEG 3350, 0.2M sodium formate, pH 7.0, soaked with 10mM thimerosal (Sigma-Aldrich) for 16 hours. The structure was refined to a resolution of 1.61 Å using Phenix.refine^32^ and by manual building in Coot^33^. Full methodology in the Supplemental Material.

### Genome-wide association studies

We performed a genetic association analysis of three loci (MOB1A, CRLF3, STK38) to test for association with 29 haematological parameters with imputed variants MAF > 0.005% and INFO score > 0.4. A significance threshold of 8.31x10^-9^ identifies associated variants located in each of the three genes by annotation with Variant Effect Predictor (VEP). Furthermore, we performed a multiple stepwise regression analysis to identify the number conditionally independent variants which represent independent association signals in each locus.

### Genetic variants in the human population

The primary data were obtained by whole-genome sequencing (WGS) from whole-blood DNA from 13,037 individuals in the NIHR BioResourceRare Diseases and 100,000 Genomes Project Pilot studies^34,35^. For Quality Control, demographics and variant calling see Karczewski *et al*^34^.

### Statistics

Sample sizes and statistical tests for each experiment are denoted in the figure legends (statistical testing was performed in Prism 8.0.1; GraphPad Software). A P value of <0.05 was considered statistically significant. *p<0.05; **p<0.01; ***p<0.005. Data are represented as mean ± S.D.

### Data Sharing

CRLF3 structures have been deposited in wwPBD under accession numbers 6RPX, 6RPY and 6RPZ. Mass spectrometry data has been deposited in PRIDE under PXD017026

For original data please contact cg384@cam.ac.uk. Additional methods used in this study are in the Supplemental Material, available on the *Blood* website.

## Results

### *Crlf3* deficiency causes an isolated reduction in platelet count

**Figure 1:**
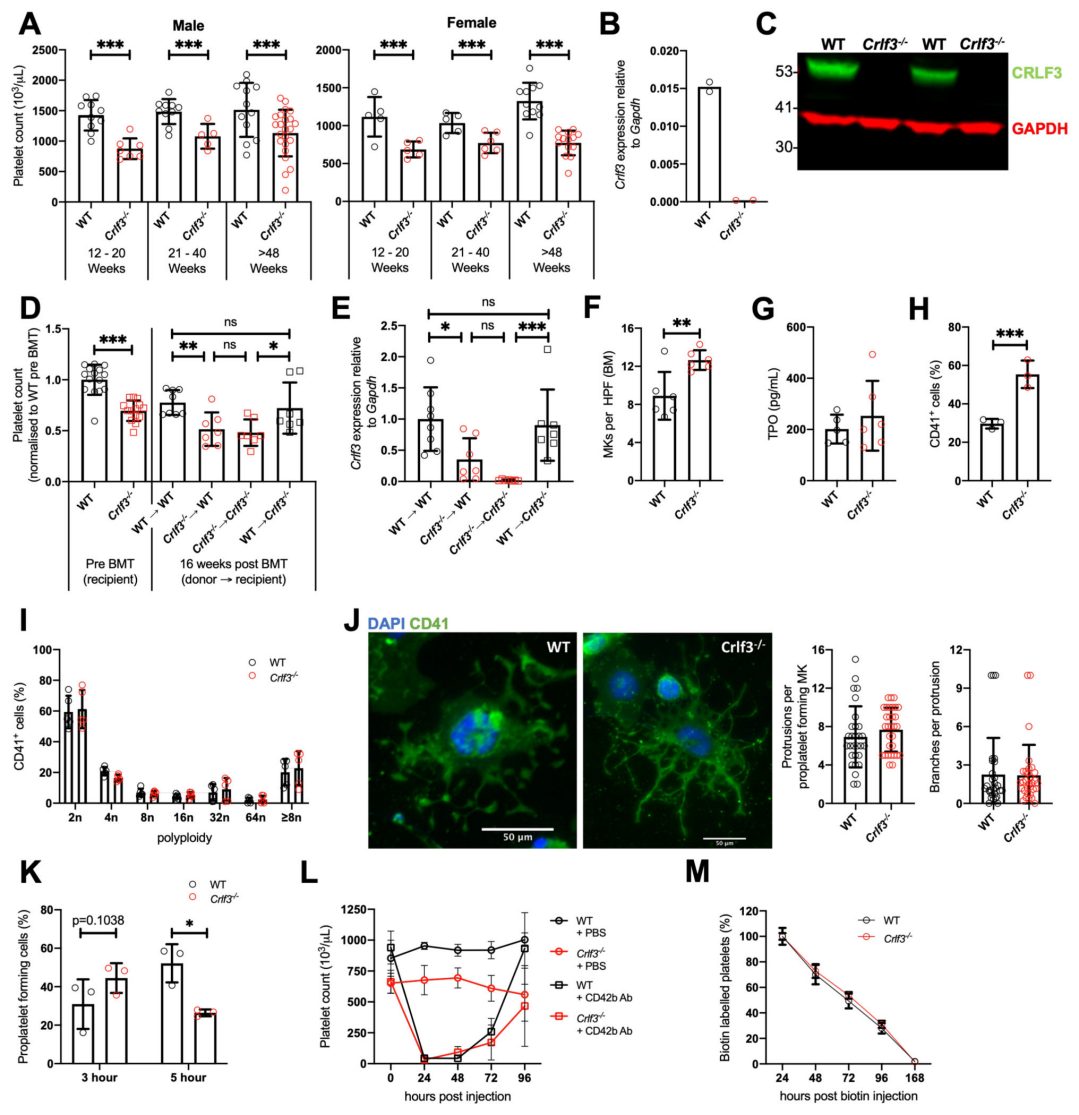
CRLF3 deficiency causes a sustained and isolated reduction in platelet count. (**A**) Platelet counts of male (*n*=5-23) and female (*n*=5-14) young (12-20 weeks), middle aged (21-40 weeks) and old (>48 weeks) control (WT, black) and *Crlf3^-/-^* (red) mice. (**B**) Expression of *Crlf3* relative to *Gapdh* mRNA determined by qRT-PCR of WT (black) and *Crlf3^-/-^* (red) isolated from *in vitro* cultured MKs (*n*=2). (**C**) Western blot of platelet lysates against CRLF3 (green) and GAPDH (red) (*n*=2). (**D**) Platelet counts pre- (on the left; *n*=15 WT/14 *Crlf3^-/-^)* and 16 weeks post-bone marrow transplantation (BMT; on the right) of control (WT, circles) and *Crlf3^-/-^* (squares) recipient mice that received either WT (black) or *Crlf3^-/-^* (red) donor cells (*n*=8 WT->WT/7 all other groups). (**E**) Chimaerism was estimated by expression of *Crlf3* relative to *Gapdh* mRNA isolated from *in vitro* cultured MKs by qRT-PCR (*n*=8 WT->WT/7 all other groups). (**F**) Quantification of MKs in H&E stained sections of control (WT, black) and *Crlf3^-/-^* (red) tibia (*n*=6). (**G**) Thrombopoietin (TPO) concentration determined by ELISA in control (WT, black) and *Crlf3^-/-^* (red) plasma (*n*=5 WT/6 *Crlf3^-/-^)*. (**H**) Percentage of CD41^+^ cells from control (WT, black) and *Crlf3^-/-^* (red) *in vitro* MK cultures (*n*= 3). (**I**) Polyploidy of *in vitro* cultured control (WT, black) and *Crlf3^-/-^* (red) MKs analysed by flow cytometry (*n*=5). (**J**) Mature *in vitro* cultured MKs were purified by BSA-gradient, seeded onto fibrinogen coated coverslips and incubated at 37°C for 5 hours to induce proplatelet formation. Fixed samples were stained with CD41 (green) and DAPI (blue), and imaged by fluorescence microscopy. Images are representative for *Crlf3^-/-^* and control (WT) proplatelet forming MKs. Scale bars are 50μm. Proplatelet morphology of control (WT, black) and *Crlf3^-/-^* (grey) MKs was assessed by blindly quantifying the number of protrusions per proplatelet forming MK and number of branches per protrusion (*n*=29 WT/31 *Crlf3^-/-^*). (**K**) *In vitro* cultured MKs were seeded onto fibrinogen coated coverslips and incubated at 37°C for 3 or 5 hours to induce proplatelet formation. After confocal microscopy, percentage of proplatelet forming MKs was determined for control (WT, black) and *Crlf3^-/-^* (red) (*n*=3). At least 460 MKs were counted in each condition. (**L**) Control (WT, black) and Crlf3-/- (red) animals were injected with PBS (circles) or anti-CD42b (0.6μg/g body weight, squares) and platelet counts determined by automated haemocytometer 0, 24, 48, 72 and 96 hours post injection (*n*=4 *Crlf3^-/-^* + CD42b Ab/3 all other groups). (**M**) Control (WT, black) and *Crlf3^-/-^* (red) mice injected with 1mg NHS-biotin and percentage of CD41^+^/Ter119^-^/streptavidin^+^ platelets was determined by flow cytometry at 24, 48, 72, 96 and 168 hours post injection. Percentage of streptavidin positive platelets at 24 hours represents 100% biotin bound platelets (*n*=5). Data represents mean ± SD. Unpaired 2-tailed Student’s *t* test (**F, G, H, J**) with correction for multiple comparisons using the Holm-Sidak method (**A**), One-way ANOVA (**D, E**) or Two-way ANOVA (**I, K, L, M**) with correction for multiple comparisons using the Holm-Sidak method. *, **, *** and ns denote *p*<0.05, *p*<0.01, *p*<0.005 and non-significant, respectively.

*Crlf3^-/-^* mice were generated as part of a genome-wide screening programme^20–22^, leading to germline deletion of *Crlf3* exon 2. Despite *Crlf3* being expressed in a large variety of tissues including all haematopoietic lineages, *Crlf3^-/-^* animals show a sustained and isolated 25-48% reduction in platelet count (p<0.005) compared to control (wild-type; WT) animals (fig.1A and Supplemental Table 1) justifying further study of this mouse strain. *Crlf3* mRNA was significantly reduced in cultured MKs from *Crlf3^-/-^*animals (fig.1B) and CRFL3 protein was undetectable in both *Crlf3^-/-^* cultured MK (fig.1C) and platelet lysates (data not shown).

To assess whether the thrombocytopenia in *Crlf3^-/-^* animals was driven by factors intrinsic to the haematopoietic compartment, we performed bone marrow (BM) transplants (BMT). Control or *Crlf3^-/-^* BM cells were transplanted into irradiated control or *Crlf3^-/-^* recipient mice. Where donor and recipient genotype were matched, the differences in platelet counts made pre-BMT remained true (fig.1D). However, when WT recipients received *Crlf3^-/-^* BM, the platelet count post-BMT decreased to comparable levels as those in *Crlf3^-/-^* recipients that received *Crlf3^-/-^* BM (p=0.9965). In contrast, when *Crlf3^-/-^* recipients received WT BM, platelet counts increased reaching levels comparable to WT recipients which received WT BM (p=0.9650). We confirmed that the post-transplant platelet count correlated with *Crlf3* expression in cultured MKs derived from recipient BM samples (fig.1E).

Next, we sought to clarify whether the thrombocytopenia was caused by decreased platelet production and/or increased platelet clearance. MK differentiation was preserved: *Crlf3^-/-^* mice have increased BM MKs (MKs per field: 12.65 ± 1.03 *Crlf3^-/-^* vs 8.90 ± 2.51 WT, p=0.0069; fig.1F and Supplemental Figure 1); TPO concentrations were only marginally increased in *Crlf3^-/-^* mice (253 ± 136 pg/mL vs 201 ± 56 pg/mL, p=0.4500; fig.1G); *Crlf3^-/-^* BM samples cultured in a suboptimal concentration of TPO showed a higher percentage of CD41 positive cells after 5 days compared to controls (55.4 ± 7.1% vs 29.7 ± 2.5%, p=0.0042; fig. 1H) and ploidy of cultured MKs was unchanged (fig.1I). Proplatelet formation was morphologically similar between *Crlf3^-/-^* and control cultured MKs (fig.1J) showing similar numbers of protrusions (p=0.2989; fig.1J, left panel) and branching (p=0.9226; fig.1J, right panel). However, proplatelet dynamics appeared altered between cultured MKs from *Crlf3^-/-^* and control animals. A greater proportion of *Crlf3^-/-^* MKs formed proplatelets 3-hours post seeding onto fibrinogen (45 ± 8% vs 31 ± 13%, p=0.1038; fig.1K and Supplemental Figure 2), whilst at 5-hours the trend was reversed (26 ± 2% vs 52 ± 10%, p=0.0164). We presume the data at 5-hours reflects proplatelet forming MKs seen at 3-hours in the *Crlf3^-/-^* sample having fragmented into platelets at 5-hours, which is supported by reduced density of *Crlf3^-/-^* MKs at 5-hours (Supplemental Figure 2). Using *in vivo* 2-photon intravital microscopy, we confirmed *Crlf3^-/-^* MKs formed long proplatelet protrusions into BM sinusoids which appeared to be no different from those seen in control animals (Video 1 and 2). Finally, we assessed platelet recovery following platelet depletion. Platelet counts in depleted *Crlf3^-/-^* and control animals recovered at an indistinguishable rate with platelet counts reaching their respective values prior to depletion in 96 hours (fig.1L). These data confirm that *Crlf3^-/-^* MKs differentiate normally and can produce platelets at least at a normal rate. The slight increase in MKs would imply a compensatory mechanism to increased platelet consumption.

We next considered whether the reduced platelet count was due to abnormal platelet function and/or clearance. *Crlf3^-/-^* mice do not display any overt bleeding phenotype. We carried out a tail bleeding assay and one *Crlf3^-/-^* animal did show increased blood loss in the early time point compared to controls and its *Crlf3^-/-^* littermate upon tail transection (Supplemental Figure 3A). We confirmed there was no gross differences in any of main platelet functions, namely adhesion, spreading, activation and thrombus formation. The expression of key platelet surface receptors/integrins was similar (p>0.05 for all tested; Supplemental Figure 3B). Platelet activation measured as fibrinogen binding and P-selectin surface expression by flow cytometry was comparable in response to different agonists (p>0.05 for all agonists at all doses; Supplemental Figure 3C). Platelet spreading onto fibrinogen was also not different (p=0.7717; Figure 3D). Thrombus formation of whole blood flowed at arterial shear rates over a collagen-coated surface was equally efficient (p=0.9809; Supplemental Figure 3E). Finally, we determined platelet lifespan by flow cytometry. We found that the gradual decrease in labelled platelets was identical in both control and *Crlf3^-/-^* animals, suggesting that platelets lifespan is unaffected (fig.1M).

### *Crlf3* deficiency leads to ineffective thrombopoiesis

**Figure 2:**
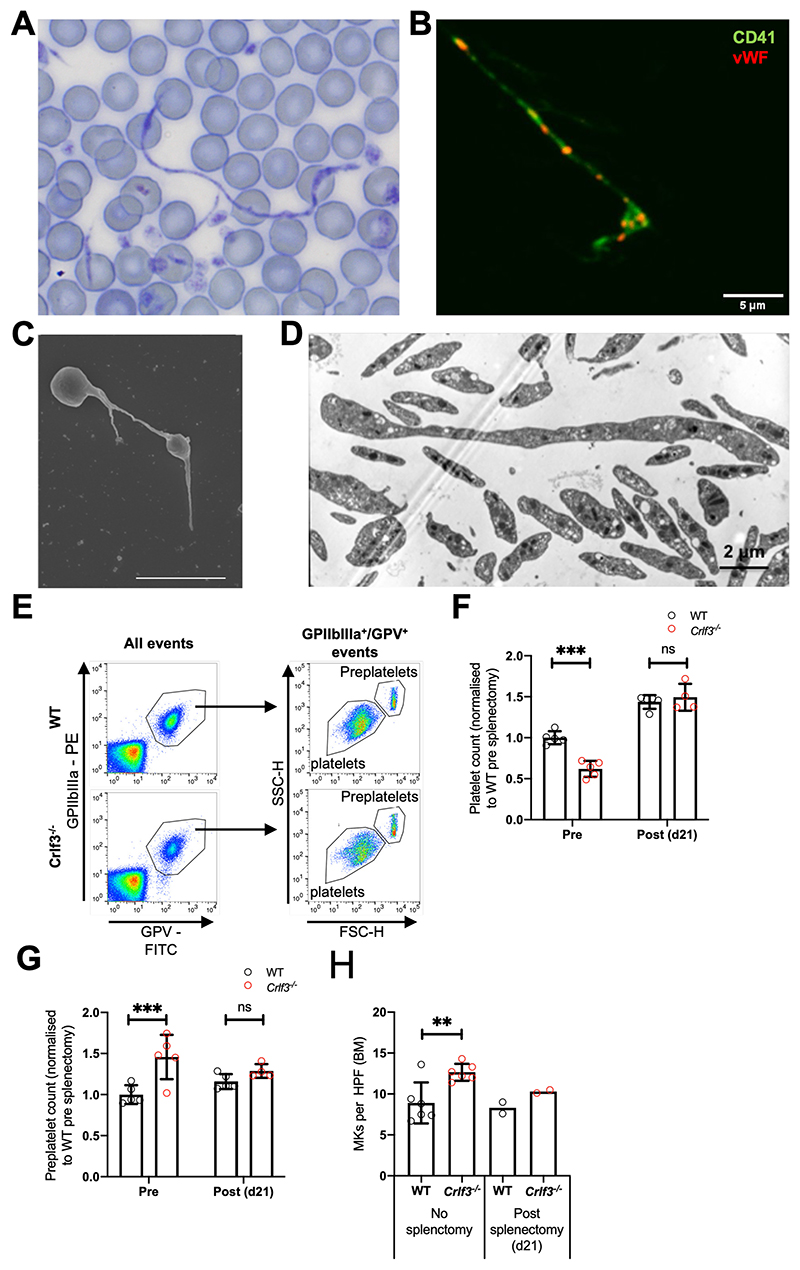
CRLF3 deficiency causes ineffective thrombopoiesis (**A**) Romanovsky-stained blood smear from *Crlf3^-/-^* mouse whole blood taken at 100x magnification under light microscopy. (**B**) *Crlf3^-/-^* blood smear stained with CD41 (green) and vWF (red) and imaged by confocal microscopy. (**C**) Washed *Crlf3^-/-^* platelets fixed and prepared for scanning or (**D**) transmission electron microscopy. Scale bars are 5μm (**B** and **C**) and 2μm (**D**). (**E**) Example flow cytometry plots to determine (**F**) platelet (GPV^+^/GPIIbIIIa^+^ events) and (**G**) preplatelet (GPV^+^/GPIIbIIIa^+^ events with larger forward scatter/side scatter than mature platelets ) counts from control (WT, black) and *Crlf3^-/-^* (red) splenectomised mice (*n*=4 *Crlf3^-/-^* post-splenectomy/5 all other groups). (**H**) Quantification of MKs in H&E stained sections of control (WT, black) and *Crlf3^-/-^* (red) tibia of non-splenectomised animals (left hand side) or 21 post splenectomy (right hand side) (*n*=6 non-splenectomised/2 splenectomised). Data represents mean ± SD (except splenectomised mice in **H**, where data represents mean). Two-way ANOVA with correction for multiple comparisons using the Holm-Sidak method (**F** and **G**), Unpaired 2-tailed Student’s *t* test (**H**). **, *** and ns denote *p*<0.01, *p*<0.005 and non-significant, respectively.

Preplatelets released into the BM sinusoids resemble proplatelet shafts, barbell platelets or giant platelets^1,2^. Preplatelets were rarely seen on the blood smears of control mice (Supplemental Figure 4A) but were easily identified on *Crlf3^-/-^* samples. Remarkably some of these were several hundred microns in length with the classical “beads on a string” appearance which has been reported before in culture but not in the peripheral circulation (fig.2A and Supplemental Figure 4A). We confirmed that these structures were preplatelets using immunofluorescence staining for specific platelet cell surface markers (CD41) and proteins contained in platelet α-granules, vWf (fig.2B), and by scanning (fig.2C and Supplemental Figure 4B) and transmission (fig.2D and Supplemental Figure 4C) electron microscopy. We hypothesized that lack of CRLF3 impairs preplatelet fission and that a proportion of these circulating “hyper-stable” preplatelets are removed from the peripheral circulation (primarily in the spleen) before they have the chance to mature into platelets. This decreases the number of new platelets produced at by each MK (ineffective thrombopoiesis). We hypothesized that splenectomy would allow the preplatelets to circulate longer, allowing them to undergo fission and correct the platelet count. We measured circulating platelet and preplatelet counts by flow cytometry pre- and post-splenectomy by a published method^3^ (fig.2E). Spleen size and weight as well as histology were comparable between *Crlf3^-/-^* and control animals (Supplementary Figure 4E). Control animals showed a slight increase in the platelet count as expected post-splenectomy (fig.2F). The platelet counts in splenectomised *Crlf3^-/-^* animals also increased post-surgery but crucially reached the same level as those seen in the control animals (p=0.4344) despite being 38% lower prior to splenectomy (p<0.0001). Preplatelets were more abundant in the *Crlf3^-/-^* animals pre-splenectomy (p=0.0010; fig.2G). Post-splenectomy, circulating preplatelets increased marginally in control animals, but decreased in *Crlf3^-/-^* animals to levels like those seen in the controls (p=0.2524; fig.2G). We postulate that splenectomy allows *Crlf3^-/-^* preplatelets to circulate for long enough to mature into platelets (switching from ineffective to effective thrombopoiesis), thereby improving the number of platelets produced per MK. In keeping with this, post-splenectomy, MK numbers in the BM of *Crlf3^-/-^* animals reduced towards levels seen in control animals (fig.2H).

### *Crlf3^-/-^* MKs contain hyper-stable polyglutamylated microtubules

**Figure 3:**
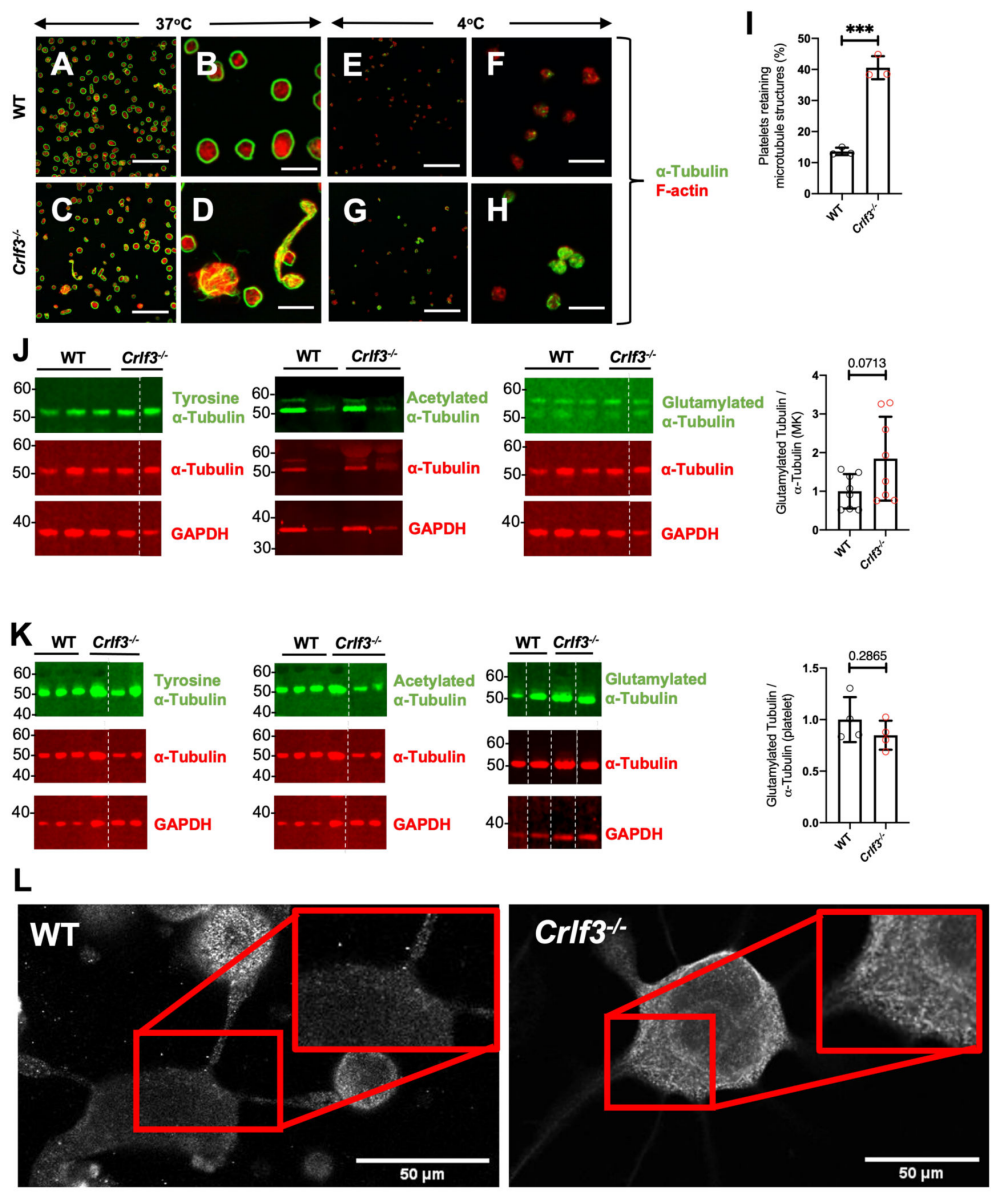
CRLF3 deficiency causes microtubule hyper-stability Washed platelets maintained at 37°C (control [WT] - **A** and **B** and *Crlf3^-/-^* - **C** and **D**) or stored at 4°C for 3 hours (control [WT] - **E** and **F** and *Crlf3^-/-^* - **G** and **H**) adhered to poly-L-lysine coated coverslips and stained for α-tubulin (green) and F-actin (red). Scale bars are 20μm (A, C, E, G) and 5 μm (B, D, F, H). (**I**) Platelets retaining microtubule structures after incubation at 4°C were determined by manual counting of images for control (WT, black) and *Crlf3^-/-^* (red) mice (*n*=3). (**J**) Representative western blots of *in vitro* cultured MK or (**K**) platelet lysates against tyrosine α-tubulin, α-tubulin and GAPDH left panel; acetylated α-tubulin, α-tubulin and GAPDH middle panel; or glutamylated α-tubulin (AG-20B-0020_upper band), α-tubulin and GAPDH right panel for control (WT, black) and *Crlf3^-/-^* (red) samples. The quantification of glutamylated α-tubulin/total tubulin (bar graphs on the right) was carried out on 8 control and 8 *Crlf3^-/-^* samples with two technical replicates. Where α-tubulin and GAPDH panels are the same for multiple tubulin modifications, membranes were stripped and re-probed between antibodies against specific tubulin modifications before finally being stripped and re-probed for α-tubulin and GAPDH. (**L**) *In vitro* cultured MKs were seeded onto fibrinogen coated coverslips and incubated at 37°C for 5 hours to induce proplatelet formation. Samples were fixed, stained for polyglutamylated α-Tubulin (AG-20B-0020) and imaged by fluorescence microscopy. Images are representative for *Crlf3^-/-^* and control (WT) proplatelet forming MKs. Scale bars are 50μm. Data represents mean ± SD. Unpaired 2-tailed Student’s (**I** and **K**) or Welch’s (**J**) *t* test. * and *** denote *p*<0.05 and *p*<0.005, respectively.

Platelet genesis is driven by microtubule assembly and re-organisation. Proplatelet formation and preplatelet release rely on microtubule formation in the proplatelet shaft, whereas preplatelet maturation into mature platelets requires tubulin bundle twisting, followed by disassembly and severing. Tubulin staining in control platelets and the majority of the mature *Crlf3^-/-^* platelets showed the classical peripheral coil (fig.3A-D). However, some *Crlf3^-/-^* platelets had disorganised tubulin, particularly in preplatelets (fig.3C-D). Most control platelets fully disassembled their microtubule coil upon cooling to 4°C, whereas a significantly larger proportion of *Crlf3^-/-^* platelets did retain at least partially some of the marginal band (41 ± 4% vs 14 ± 1%, p=0.0003; fig.3E-I). Microtubule stability is influenced by post-translational modifications (PTMs) such as tyrosination, acetylation or glutamylation^26–28^. We saw no difference in the total tubulin content (p=0.1978), tubulin tyrosination (p=0.7218) and tubulin acetylation (p=0.1312) of cultured *Crlf3^-/-^* MKs, normalised to total tubulin content (fig.3J and Supplemental Figure 5A-C). However, polyglutamylated tubulin content appeared increased in cultured *Crlf3^-/-^* MKs, albeit not reaching significance (1.85-fold increase; p=0.0713; fig. 3J). We therefore probed MK samples with an alternative antibody against polyglutamylated tubulin and showed a similar small increase in polyglutamylated tubulin content (1.66-fold; p=0.0050; Supplemental Figure 5D). Platelet tubulin content and modified tubulins were also analysed but failed to reveal any significant differences, however there was a trend towards increased tyrosinated tubulin in platelets (1.47-fold increase; p=0.0639; fig.3K and Supplemental Figure 5A-C). We postulate that polyglutamylated tubulin was unchanged in platelets as those would be most likely arising from MKs with the lowest level of glutamylation allowing for prompt proplatelet maturation. Using immunofluorescence, we showed bundles of glutamylated tubulin leading towards the proplatelet shafts in some *Crlf3^-/-^* MKs (fig.3L) but this was not the case in all cells analysed (Supplemental Figure 5E).

### CRLF3 interacts with STK38 and its absence leads to increased MOB1 phosphorylation

**Figure 4:**
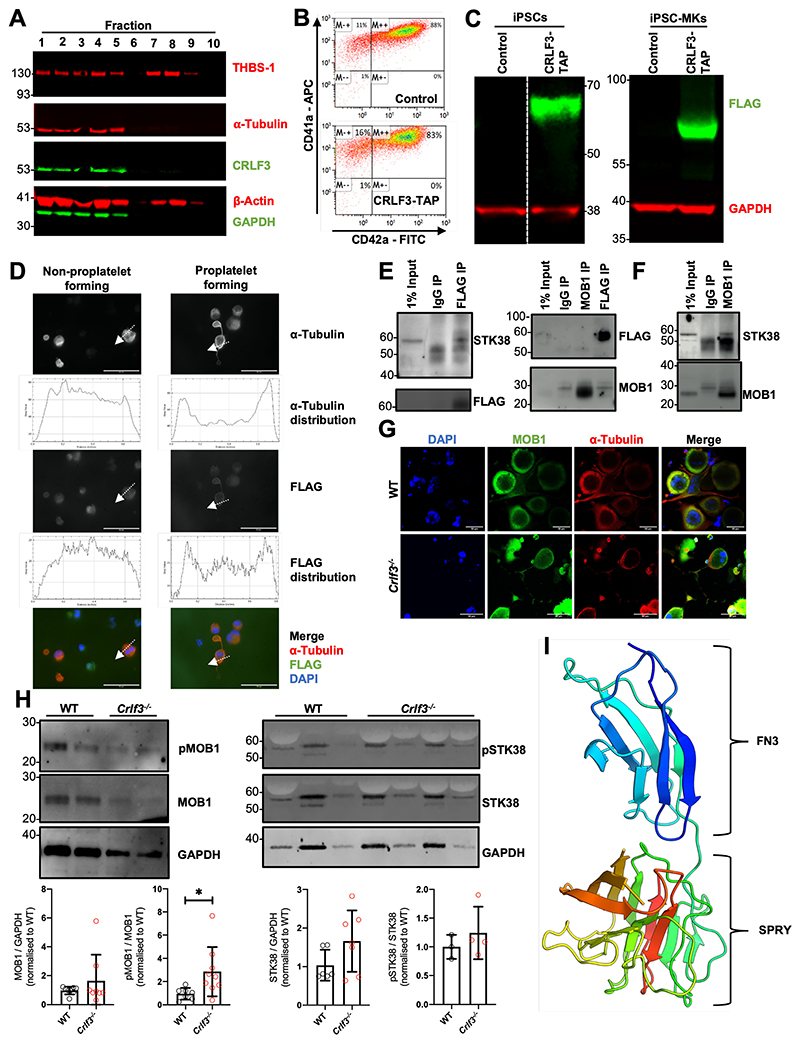
CRLF3 interacts with the Hippo pathway (**A**) Western blot of sucrose gradient centrifugation fractionated human platelets probed with antibodies against α-tubulin, β-actin, thrombospondin (THBS-1), GAPDH and CRLF3. Fractions 1-5 represent cytoskeletal proteins (enriched for α-tubulin and β-actin), whereas fractions 7 and 8 represent granular proteins (enriched for the THBS-1). (**B**) Representative flow cytometry plots of CRLF3-TAP tagged and control forward programmed iPSC-MKs stained with CD41a and CD42a. (**C**) Western blot of CRLF3-TAP tagged and control iPSCs and iPSC-MKs probed with antibodies against FLAG (green) and GAPDH (red). (**D**) CRLF3-TAG tagged iPSC-MKs were seeded onto fibrinogen coated coverslips and incubated at 37°C for 24 hours to induce proplatelet formation. Samples were fixed, stained with α-tubulin (red), FLAG (green) and DAPI (blue), and imaged by fluorescence microscopy. Sub-cellular distribution of α-tubulin and FLAG staining in round and proplatelet forming CRLF3-TAP iPSC-MKs was determined across a section of the MKs along the indicated arrow using ImageJ. Scale bars are 10μm. (**E**) CRLF3-TAP and (**F**) control iPSC-MKs were lysed and immunoprecipitated with antibodies against FLAG, MOB1 and IgG. Precipitated lysates were then probed for STK38, MOB1 and FLAG by western blot. (**G**) *In vitro* cultured MKs were seeded onto fibrinogen coated coverslips and incubated at 37°C for 5 hours to induce proplatelet formation. Samples were fixed, stained for MOB1, α-Tubulin and DAPI and imaged by fluorescence microscopy. Images are representative for *Crlf3^-/-^* and control (WT) proplatelet forming MKs. (**H**) Western blot of in *vitro* cultured MKs probed with antibodies against pMOB1, MOB1 and GAPDH (left panel; n=8 MOB1/GAPGH and 4 pMOB1/GAPDH) and pSTK38, STK38 and GAPDH (right panel; n=3 STK38/GAPDH, 3 *Crlf3^-/-^* and 4 WT pSTK38/GAPDH). (**I**) 3D structure of CRLF3 construct 3 (residue 174 to end) solved by experimental phasing. Domains are labelled. Molecular graphics prepared using PyMOL. FN3 = fibronectin type 3. Data represents mean ± SD. Unpaired 2-tailed Student’s *t* test. * denotes *p*<0.05.

To gain a mechanistic understanding CRLF3’s role in tubulin glutamylation, we switched over to a human cellular system. First, we sought to identify the CRLF3’s cellular localisation and protein partners in relevant cells e.g. platelets and MKs. Human platelet lysates were sub-fractionated by sucrose gradient centrifugation^36^. Western blot analysis clearly showed enrichment of CRLF3 in the sub-fractions containing cytoskeletal proteins, particularly α-tubulin (fig.4A). To refine CRLF3’s localisation and perform pull down experiments, we used a human induced pluripotent stem cell (iPSC)-based system. We inserted a TAP-tag^37^ at the 3’ end of the endogenous *CRLF3* gene in iPSCs. Tagged and control iPSCs were differentiated into highly pure populations of MKs (fig.4B) by forward programming^27^. The expression of CRLF3-TAP was confirmed in both tagged iPSCs and their MK progeny (iPSC-MKs; fig.4C). In non-proplatelet forming iPSC-MKs CRLF3-TAP showed a diffuse mainly cytoplasmic pattern. By contrast in proplatelet forming iPSC-MKs, CRLF3-TAP appears to redistribute to the plasma membrane (fig.4D). We went on to perform mass spectrometry on anti-FLAG immunoprecipitation samples from CRLF3-TAP and control iPSC-MKs and several candidate interacting proteins were identified (Supplemental Table 2). One candidate interactor was STK38, a member of a group of NDR kinases known to interact with MOB1^38^. MOB1 is a key member of the Hippo pathway^39^ and a protein that has been shown to influence tubulin stability through PTM^40^. MOB1 has previously been shown to interact with CRLF3 in HEK cells treated with okadaic acid^41,42^. We performed anti-FLAG immunoprecipitation followed by western blotting on okadaic acid treated CRLF3-tagged iPSC-MKs and confirmed the interaction between CRLF3 and STK38 (fig.4E). We could not show evidence of an interaction between CRLF3 and MOB1 in either the forward or reverse pulldowns (fig. 4E), however in anti-MOB1 immunoprecipitated control iPSC-MKs, we confirmed the interaction between MOB1 and STK38 (fig.4F). We did not see a difference in MOB1 localisation (fig.4G) or total quantity of MOB1 (p=0.3337; fig.4H) in *Crlf3^-/-^* MKs. However, we saw increased phosphorylation of MOB1 (>2.5-fold, p=0.0286; fig.4H and Supplemental Figure 6). Since we established an interaction between CRLF3 and STK38 in MKs, we looked at CRLF3’s influence on STK38 protein. We saw a small (≈1.5 fold) non-significant increase in total quantity of STK38 (p=0.1112; fig.4H) in *Crlf3^-/-^* MKs but STK38 phosphorylation was unchanged (p=0.4398).

Finally, we sought to determine the crystal structure of CRLF3. We were only successful in expressing the C-terminal portion of CRLF3 (amino acid 174 to 442) at sufficient levels for crystallography. Crystals were successfully obtained using the sitting drop vapour diffusion method. Native data were collected to a resolution of 1.61 Å, and the structure was solved by Hg-single-wavelength anomalous diffraction phasing. This revealed a 3D structure containing two known protein binding domains, a fibronectin type III (FN3) domain (residues 179 to 273) and a SPRY domain (residues 274-442) (fig.4I). Crystallographic statistics can be found in Supplemental Table 3. The native structure, refined to R_work_=0.177 and R_free_=0.201, has been deposited at the worldwide PDB with ID 6RPX.

### CRLF3 in human thrombopoiesis

**Figure 5:**
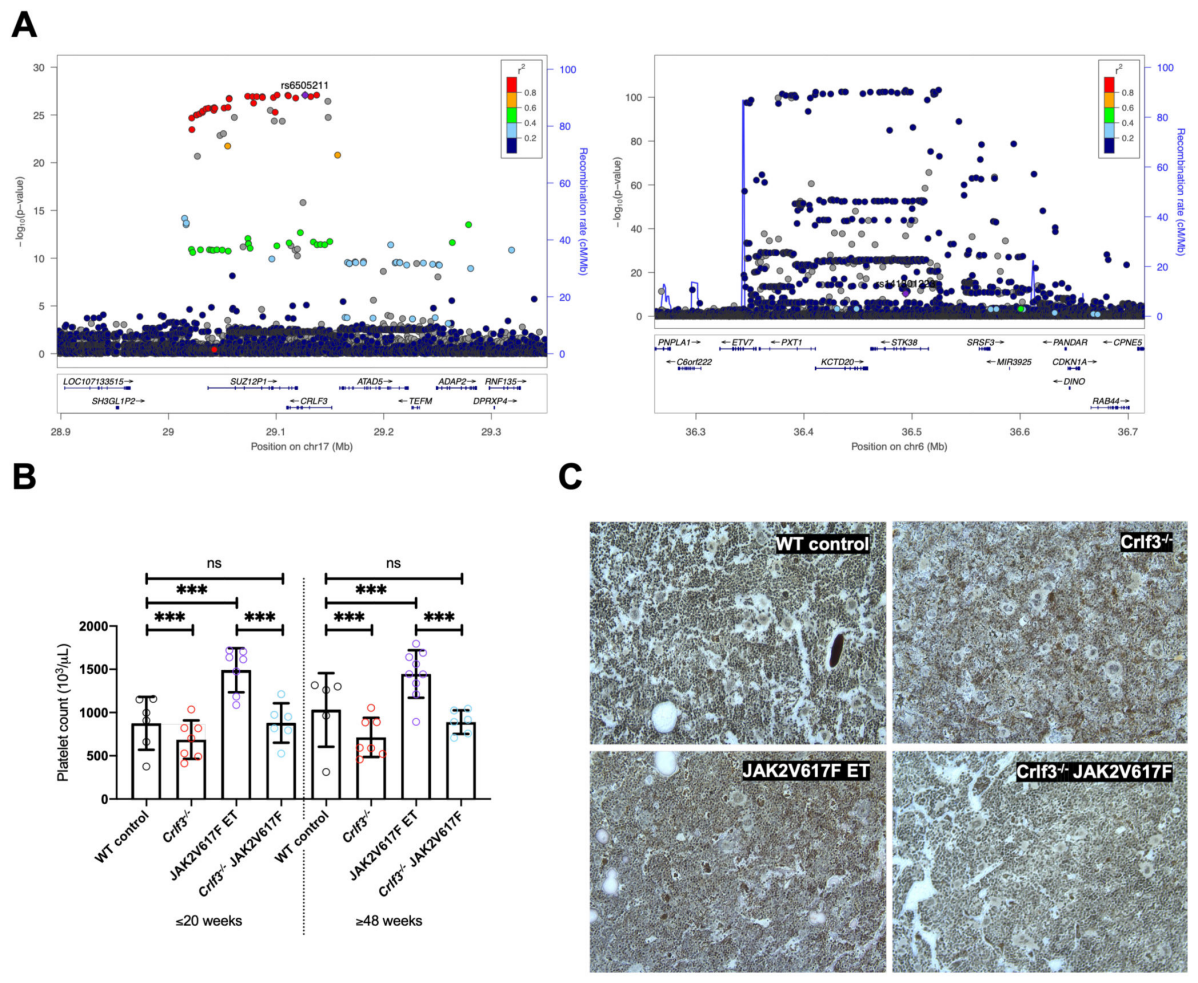
CRLF3 regulates platelet traits in humans and is a therapeutic target for Essential Thrombocythaemia (**A**) Locuszoom of CRLF3 (left) and STK38 (right) showing variants associated with Platelet Distribution Width (PDW) and Mean Platelet Volume (MPV), respectively. The conditionally independent variant is indicated by a purple diamond, LD values (r2) with this variant are indicated by dot colours according to the legend above. The CRLF3 locuszoom plot shows the conditionally independent variant (rs6505211, -log10P: 27.1, MAF: 17.6%) is in high LD with a number of variants which are significantly associated with PDW. In the case of STK38 the locuszoom plot indicates that the conditionally independent variant (rs141301223 -log10P: 10.4, MAF: 0.041%) is not in high LD with nearby variants (common for rare variant associations). (**B**) Platelet counts from young (≤20-week-old) and old (≥48-week-old) female WT control (black; *n*=6 young/5 old), *Crlf3^-/-^* (red; *n*=7 young/7 old), JAK2V617F ET (purple; *n*=7 young/9 old) and *Crlf3^-/-^* JAK2V617F (blue; *n*=6 young/6 old) mice. (**C**) Fixed tibia sections stained with Gömöri’s reticulin silver stain and imaged by lightX microscopy at 20x magnification. Images are representative of 3 mice per genotype. Data represents mean ± SD. Two-way ANOVA with correction for multiple comparisons using the Holm-Sidak method. *** and ns denote p<0.005 and not significant, respectively.

We used a genetic approach to look for evidence that CRLF3 and its partners play a role in human thrombopoiesis. Using the imputed genotype data from 403,112 European ancestry participants in UK Biobank, we performed univariable association analyses between 29 haematological traits and genetic variants in the loci containing CRLF3, MOB1A and STK38. Our analyses identified significant (-log_10_ P>8.08) associations with platelet distribution width (PDW) in the CRLF3 locus (fig.5A left panel), of which the variant with the strongest evidence for association (rs6505211; purple diamond) is in the gene body. This variant was in high LD (r^2^>0.8) with the variant exhibiting the strongest evidence for association with platelet distribution width (PDW) but also lymphocyte percentage of total white blood cells (LYMPH%). We also identified associations with variants in STK38, which were significantly associated with mean platelet volume (MPV) (fig.5A right panel) and identified a variant in MOB1A significantly associated with platelet count (Supplemental Table 4). The latter variant is not associated with other haematological traits. This data therefore suggests a role for all 3 genes in thrombopoiesis without necessarily implying that there is a mechanistic link between them. Amongst a collection of 59,464 individuals comprising probands affected with rare disorders for whom Human Platelet Ontology (HPO) terms are available and their first-degree relatives, we identified 27 who were heterozygous for severe impact variants in *CRLF3*. None had HPO terms suggesting a haematological phenotype (Supplemental Table 5). No homozygous individuals for severe impact variants were identified in this cohort. Five individuals (including 2 siblings) were identified who were homozygous for missense variants but again, none had a haematological phenotype. The severe variants were shown to be low frequency in gnomAD^34,43^. The missense variants were inputted into the crystal structure described above. Ala279Val and Asn410Asp have a minor allele frequency (MAF) of 2.4 x 10^-5^ and 4.7 x 10^-4^ in gnomAD, respectively, but are minor changes on the surface of the protein. Leu389Pro has a MAF of 15%, and together with the last variant Thr392Ile, is part of a disordered loop, (amino acid 387-398), again, on the surface of the protein.

### CRLF3 is a potential therapeutic target for Essential Thrombocythaemia

We postulated that the specific effect of CRLF3 deficiency on platelet count, would make CRLF3 a potential therapeutic target in ET. As a proof of principle, we crossbred *Crlf3^-/-^* mice with a previously published inducible knock-in mouse model of ET driven by the JAK2V617F mutation^44^. The breeding strategy described in Supplemental Figure 7 generated 4 groups of animals: WT control, *Crlf3^-/-^*, JAK2V617F ET and *Crlf3^-/-^* JAK2V617F mice. We assessed the platelet counts in these 4 groups of mice at both young (≤20 weeks) and old (≥48 weeks) age and showed that ablation of *Crlf3* in JAK2V627F ET mice normalised the platelet count to the levels seen in control mice (fig.5B). Crucially, we showed that all other blood counts were unaffected (Supplemental Table 6). Platelet function analysis in all 4 groups of mice showed no differences. No additional clinical findings were made in the *Crlf3^-/-^* JAK2V627F mice, including no evidence of bone marrow fibrosis (fig.5C) or changes in spleen size/weight (data not shown).

## Discussion

Thrombopoiesis is classically described as a two-stage process comprising first MK differentiation and maturation from HSCs followed by the actual process of platelet release. Proplatelet formation, which has been observed *in vivo* ^1,2^, is the broadly accepted mechanism by which MKs release platelets, although some authors have argued that MK fragmentation^45^ or membrane budding^46^ may constitute alternative mechanisms. Proplatelet fragments detach from MKs forming long “beads on a string” structures (preplatelets) that become mature discoid platelets^3^. In this manuscript, we show that preplatelet fission is critical for regulating platelet production and is the third and final stage of platelet production. We show that mice deficient in *Crlf3* have reduced platelet counts due to *ineffective thrombopoiesis* whereby slowed maturation of circulating preplatelets leads to their removal by the spleen, reducing the number of circulating platelets. This data clearly suggests the central role of the proplatelet formation and subsequent preplatelet fission in platelet biogenesis as opposed to MK fragmentation or blebbing.

It has long been known that proplatelet formation is a process in which cytoskeletal proteins play a key role. Our data suggests that CRLF3 deficiency may exert its effect on preplatelet maturation through increasing tubulin stability. We show small changes in tubulin polyglutamylation in the primary mouse MKs which may, in part, explain these observations. This would need to be confirmed in MKs derived from cell lines, rather than primary MKs, to allow for detailed protein and PTMs studies to be carried out. We should note that it has been shown using cell line models that tubulin polyglutamylation has been shown to promote proplatelet-like extensions in CHO cells^47^ and affects the localisation of motor protein in MKs^48^. In keeping with this, we observed an increased rate of proplatelet formation in *Crlf3^-/-^* MKs. Tubulin’s C-terminal tail is subjected to diverse PTMs which vary between cell-type and intracellular localisation^49^. This allows fine spatial and temporal control of microtubule function by modifying the binding of microtubule-associated proteins (including microtubule severing enzymes). Although, the key enzymes involved in controlling tubulin glutamylation and severing are expressed equally between *Crlf3^-/-^* and control MKs (Supplemental Table 7), we postulate that the increase in tubulin glutamylation observed in the *Crlf3^-/-^* MKs is such that it may subsequently affect tubulin severing in the *Crlf3^-/-^* preplatelets, thereby preventing their maturation. This hypothesis deserves further studies, potentially using genetically modified MKs derived from cell lines enabling fine tuning of tubulin polyglutamylation to study proplatelet formation and maturation dynamics.

Previously, direct interaction between CRLF3 and MOB1 has been reported^41,42^. MOB1 acts as a co-activator of NDR kinases, including STK38^38^. MOB1 phosphorylation increases its binding to and activation of STK38 ^39^ and localises the complex to the plasma membrane, especially at sites of pseudopodia/cytoplasmic extensions^50^. MOB1 is known to affect tubulin stability through regulating acetylation and thereby cytokinesis^40^. In this study we confirm that, in MKs, STK38 associates with both MOB1 and CRLF3, but we did not show a direct interaction between CRLF3 and MOB1. However, MOB1 phosphorylation was increased in *Crlf3^-/-^* MKs and we also show that CRLF3 relocates to the membrane, but only in proplatelet forming MKs. Taken together these data strongly support the need for further studies in MKs to provide definitive proof that MOB1 influences PTMs of tubulin in MKs and thereby influences proplatelet formation and maturation dynamics and ultimately circulating platelet numbers.

Most ET patients are administered non-specific cytoreductive therapies to lower their platelet count. Hydroxyurea, the most commonly prescribed agent, causes anaemia, leukopenia or skin ulcers^51,52^ in up to 20% of patients and concerns about an associated leukaemic risk remain^53,54^. Anagrelide, the second most commonly prescribed, is associated with a 3-fold increased risk of myelofibrosis compared to hydroxyurea^55^. Identification of a novel biological pathway that, when targeted, could specifically reduce platelet count is very promising for the treatment of ET. Targeting CRLF3 may allow specific reductions in platelet count by acting at the level of preplatelet maturation, as evidenced with our *Crlf3^-/-^* JAK2V617F ET murine model. These mice showed sustained and isolated normalisation of their platelet count without increased bone marrow fibrosis or leukaemic transformation. An increased understanding of CRLF3’s role in other cell types, its structure and its structural relationship with partner proteins, such as STK38 and MOB1, as well as defining how CRLF3 interacts with tubulin post-translation modifications, could potentially generate drugs that would complement (or supersede) the current non-specific cytoreductive agents. Further studies of the role of CRLF3 should also focus in the human system to confirm the findings of the murine studies presented here.

## Supplementary Material

Supplementary Material

Supplementary tables 2, 4, 5, and 7

Supplementary Video 1

Supplementary Video 2

## Key Points

Cytokine receptor-like factor 3 (CRLF3) deficiency causes an isolated and sustained reduction in platelet count in mice.

CRLF3 is a potential therapeutic target for thrombocythaemia.
