# Supplementary material for: CRLF3 plays a key role in the final stage of platelet genesis and is a potential therapeutic target for thrombocythaemia
Source: Blood. Author manuscript; Available in PMC 2023 Jun 16. (PMC7614665; doi:10.1182/blood.2021013113)
Supplement: Supplementary Material [file EMS176309-supplement-Supplementary_Material.pdf]

Supplemental Data

Supplemental Methods

Generation of *Crlf3*$^{-/-}$ mice

The generation of *Crlf3*$^{-/-}$ mice is illustrated in Supplemental Figure 7. The ES cell clone used was EPD0033_3_C11 and was selected based on neomycin resistance and β-galactosidase expression confirming *Crlf3*$^{tm1a(KOMP)Wtsi}$ allele expression. Mice were generated according to standard techniques and after germline transmission of the *Crlf3*$^{tm1a(KOMP)Wtsi}$ allele, exon 2 of *Crlf3* was excised by Cre-mediated recombination by crossing with *Hprt*$^{Tg(CMV-cre)Brd}$ mice, producing the mutant *Crlf3*$^{tm1b(KOMP)Wtsi}$ allele. After confirmation of allele conversion by PCR, chimeric mice carrying the *Crlf3*$^{tm1b(KOMP)Wtsi}$ allele were backcrossed to C57BL/6 mice.

Blood and tissue analysis

Blood smears were created by pushing whole venous blood across a microscope slide and leaving to air dry overnight. Blood smears were then stained with Rapid Romanowsky staining kit (TCS biosciences) following the manufacturer's instructions. For confocal microscopy, blood smears were washed with 50mM ammonium chloride to remove red cells, then remaining cells were permeabilised with PBS containing 0.1% w/v saponin and 0.2% w/v gelatin (both Sigma-Aldrich). Samples were incubated with primary antibodies against CD41 (133902; BioLegend) and vWF (A0082; DakoCytomation). Samples were washed before incubating with appropriate secondary antibodies (Alexa Fluor 488 Goat anti-rat IgG (A-11006) or Alexa Fluor 555 Goat anti-rabbit IgG (A-21428); both Invitrogen). Samples were again washed before glass coverslips were mounted using hydromount (National Diagnostics) and images were acquired using a Leica Sp5 inverted confocal microscope with the 63x immersion-oil objective and the Leica LAS 2.1 software.

Plasma thrombopoietin (TPO) levels were measured with the Quantikine Mouse TPO ELISA kit (R&D Systems).

For histologic analysis, mice were examined after sacrifice. Specimens were collected into 10% neutral buffered formalin solution (Sigma-Aldrich). Fixed tibia and spleens were embedded in paraffin and sections cut, deparaffinised and rehydrated. Sections were then stained with

Haematoxylin and Eosin stain or Gömöri's reticulin silver stain for reticulin fibres. After staining, images were acquired using an Axiovert 40 CFL or Olympus microscope and AxioVision 40 version 4.8.2.0 software (both Zeiss) and MKs blindly quantified manually. A Pixera 600ES camera was used for digital photomicroscopy of the blood and bone marrow.

Expression array and qRT-PCR

RNA was extracted from purified *in vitro* cultured MKs using RNeasy plus mini Kit (Qiagen). Complementary (c) DNA was made using SuperScript III First Strand Kit (Invitrogen) starting from 100ng total RNA and using oligo-dT as reverse transcription primers. Expression levels were quantified using Illumina Mus 6v2 expression chip. Data processing and analysis were performed as described elsewhere[1]. Quantitative polymerase chain reaction (qPCR) of the murine genes *Crlf3* and *Gapdh* was carried out using specific primers that had previously been tested for optimal efficiency, Brilliant II SYBR green chemistry (Agilent Technologies) and Mx3000P Real-Time PCR system (Stratagene). The analysis of gene expression was performed by the ΔCt method, where:

ΔCt = Ct target gene (*Crlf3*) – Ct housekeeping gene (*Gapdh*)

Relative gene expression was expressed as $2^{-\Delta Ct}$.

Crlf3 specific primers:

Forward: 5'-GGGTGTTCTCTACTCCAGCG-3'

Reverse: 5'-GCCATTCTGCCTTTCTGCAC-3'

Gapdh specific primers:

Forward: 5'-CCCTTAAGAGGGATGCTGCC-3'

Reverse: 5'- TACGGCCAAATCCGTTCACA-3'

Western blot

Total cellular lysates of washed platelets, iPSCs and iPSC-MKs were prepared by direct cellular lysis with RIPA buffer (150mM sodium chloride, 60mM Tris pH 8.0, 0.6% w/v sodium deoxycholate, 0.1% w/v sodium dodecyl sulfate, 1% v/v NP-40) supplemented with protease (Roche) and phosphatase (Sigma-Aldrich) inhibitors, incubation on ice for 30 min and followed by centrifugation at 13,000*g* at 4°C for 5 min. Fractionated platelet samples were prepared as previously described[2]. For immunoprecipitation (IP) samples, iPSCs were harvested at D20 of MK forward programming. iPSC-

MKs were centrifuged for 8 min at 120 g, washed three times in ice cold PBS and snap frozen on dry ice. Samples were then resuspended in 10 mM Tris-HCl pH 8, 150 mM NCl, 0.5 % NP-40 and protein content was quantified by BCA assay (Thermo). 50 µL Protein G Dynabeads (Invitrogen) were washed x3 in PBS 0.01% Tween-20. Samples were resuspended in 50 µL PBS 0.01% Tween-20 and incubated for 40 min at RT on an orbital rotator with 1 µg of antibody. The beads were washed x3 in PBS 0.01% Tween-20 and added to cell lysate at 1 ml/mL. Samples were rotated at 225 rpm at 4°C overnight. Supernatants were stored and beads were washed x3 in PBS 0.01% Tween-20 with 1X protease inhibitors (cOmplele mini EDTA free, Roche) and 1X PhosSTOP (Roche). They were transferred to a new eppendorf on the last wash and resuspended in 30 µL MPER (Thermo) 1:4 NuPAGE sample buffer (Invitrogen) with 1:10 reducing agent (Invitrogen). Denaturation was carried out for 10 min at 70°C. Supernatant was collected from beads and loaded on a Western Blot as below.

Samples were diluted 1:1 with 2x Laemmli sample buffer (125mM Tris pH 6.8, 20% v/v glycerol, 4% w/v sodium dodecyl sulfate, 10% v/v β-mercaptoethanol, 0.002% w/v bromophenol blue) and incubated at 95°C for 5 min to denature proteins. Proteins were resolved by sodium dodecyl sulfate–polyacrylamide gel electrophoresis under reducing conditions and transferred to PVDF membrane (Millipore) and probed by the following antibodies. Membranes were imaged with Odyssey Fc OFC-1095 version 1.0.36 imaging system. Expression was quantified using Image Studio Lite.

The following primary antibodies were used: anti-CRLF3 (HPA007596; Atlas antibodies), anti-GAPDH (2118; Cell Signalling Technology), anti-α-tubulin (T5168; Sigma-Aldrich and GTX102078; GeneTex), anti-tyrosine tubulin (T9028; Sigma-Aldrich), anti-glutamylated α-tubulin (AG-20B-0020 and AG-25B-0030; AdipoGen Life Sciences), anti-acetylated α-tubulin (sc23950; Santa Cruz Biotechnology), anti-FLAG (F1804; Sigma-Aldrich), anti-β-actin (A1978; Sigma-Aldrich), anti- STK38 (ab56977; Abcam), anti-pSTK38 (SPC-1035; StressMarq), anti-MOB1A (ab221988; Abcam), anti-pMOB1 (8699S, Cell Signalling Technology) and anti-human thrombospondin (ab1823; Abcam). Where α-tubulin or modified α-tubulin blots presented as a double band in MK samples, analysis was conducted on the band corresponding to the band seen in platelet samples.

For the measurement of the post-translational modification of tubulin, we first measured the polyglutamylated/acetylated/tyrosinated tubulin content. Following this, the membranes were stripped and total tubulin and GAPDH (loading control) content were measured concurrently. Before re-probing, stripped membranes were checked to be blank across all lanes. This method allowed us

to measure total tubulin content (vs GAPDH) as well as the ratio of modified tubulin vs total tubulin on the same samples. In some blots we measured one type of tubulin modification, then another after stripping the membrane and finally total tubulin and GAPDH.

In vitro proplatelet formation

Mature MKs were resuspended in culture medium supplemented with 100U/mL heparin sodium (Wockhardt UK Ltd) and seeded onto glass coverslips coated with 20ng/mL fibrinogen (Enzyme Research). Cells were incubated at 37°C under 5% CO_2 for either 3 or 5 hours. Attached MKs were fixed with 10% neutral buffered formalin solution and washed in PBS. Fixed MKs were stained with antibodies against CD41 (133902; BioLegend or sc-19963; Santa Cruz Biotechnology), F-actin (Alexa Flour 555 Phalloidin (A34055); Invitrogen), polyglutamylated tubulin (AG-20B-0020 or AG-25B-0030; AdipoGen Life Sciences), MOB1A (ab221988, Abcam), α-tubulin (T5168, Sigma-Aldrich), β -tubulin (ab21057; Abcam), and the nuclear stain DAPI (Sigma-Aldrich). Images were acquired using a Leica Sp5 inverted confocal microscope with the 20x objective and the Leica LAS 2.1 software or a Zeiss LSM 880 MP Airyscan inverted confocal microscope with the 20x objective and Zeiss Zen software. For proplatelet dynamics, proplatelet forming MKs were counted against the total number of MKs to calculate the percentage of proplatelet forming MKs. At least 460 MKs were counted for each condition.

Bone marrow transplantation

Recipients were sub-lethally irradiated with a split dose of 950 cGY, separated by >4 hours. 5×10^6 WT or *Crlf3*$^{-/-}$ bone marrow (BM) cells were injected into WT or *Crlf3*$^{-/-}$ recipient mice via the tail vein. Peripheral venous blood was obtained to determine platelet counts using a Woodley ABC blood counter (Woodley). Donor chimaerism was determined by *Crlf3* expression by qRT-PCR of *in vitro* cultured MKs derived from recipient BM cells taken at termination of the experiment.

Blood loss after tail transection

Experiments were performed as per Evans et al, 2021[3]

Electron Microscopy

For transmission electron microscopy (TEM), washed platelets fixed with 2.5% glutaraldehyde in 50mM cacodylate buffer (pH 7.2). After embedding in epon 812, ultra-thin sections were generated and stained with 2% uranyl acetate and lead citrate. Samples were visualised with a Zeiss EM900 microscope.

For scanning electron microscopy (SEM), washed platelets were resuspended in 0.85% sodium chloride, diluted 1:1 with 0.4% glutaraldehyde in 0.1M sodium cacodylate pH 7.4 and incubated at room temperature. Platelets were then pelleted at 1,000*g* and resuspended in fixation buffer (2% glutaraldehyde, 2% formaldehyde, 0.05M sodium cacodylate, pH 7.4). Platelets were rinsed in deionised water (DIW) and incubated in 1% osmium ferricyanide for 4 days, rinsed again in DIW and incubated in 2% uranyl acetate in 0.05M Maleate buffer (pH 5.5) for 4 days at 4°C. Platelets were resuspended in DIW and attached to poly-L-lysine coated glass coverslips. After unattached platelets were removed, samples were quench frozen in melting propane cooled in liquid nitrogen, then dried overnight in a Quorum K775 freeze dryer. The dry coverslips were attached to 13mm Cambridge SEM stubs with colloidal silver DAG sputter coated with 10 nm of iridium and viewed in a FEI Verios 460L using a secondary electron detector.

Tandem Affinity Purification (TAP)-tagging CRLF3 in induced pluripotent stem cells (iPSCs)

Tandem Affinity Purification (TAP)-tagging of CRLF3 at the C-terminus was performed by homology directed repair (HDR) by CRISPR/Cas9. CRISPR ID 1146192702 was picked from www.sanger.ac.uk/htgt/wge/ [4]. Single-guide RNA (sgRNA) expression constructs were generated using the plasmid-based procedure as described by Ran, *et al.*[5] and cloned into the pSpCas9(BB)-2A-GFP plasmid (Addgene).

A donor vector containing 1Kb homology arms surrounding the TAP-tag[6] in a pBS KS vector backbone (Addgene) was created by Gibson Assembly following manufacturer's instructions (Gibson Assembly Cloning Kit; New England Biosciences).

sgRNA/Cas9-GFP plasmid and TAP-tag donor vector DNA were introduced into Qolg (HipSci line HPSI1113i-qolg_3 (http://www.hipsci.org/)) induced pluripotent stem cells (iPSCs) in a single cell solution by nucleofection using Amaxa Human Stem Cell Nucleofector Kit 2 and an Amaxa Nucleofector II (both Lonza), following manufacturer's instructions. 48 hours after nucleofection, cells were sorted based on GFP expression by flow assisted cell sorting. GFP positive iPSCs were

seeded individually to laminin 521 (Biolamina) coated 96 well tissue culture plates and maintained in Essential 8 (E8) medium (Gibco) supplemented with 100U/ml pen/strep (PAA Laboratories) for 5 days then maintained as colonies in E8 medium on truncated vitronectin (VTN-N; ThermoFisher Scientific) coated tissue culture plastic plates (Corning) until needed.

Mass Spectrometry and LC-MS/MS analysis

$50x10^6$ TAP-tagged or untagged iPSC-MKs were dissociated to a single cell solution, washed 3 times in PBS then snap frozen and stored at -80^oC until needed. Frozen pellets were then lysed in lysis buffer (10mM Tris hydrochloride, 150mM sodium chloride, 0.1% NP-40), centrifuged at 13,000rpm for 15 minutes and lysates collected. Proteins interacting with TAP-tagged CRLF3 were then collected by incubating the lysates with magnetic beads (Dynal protein G; Invitrogen) coated with anti-FLAG antibody (anti-FLAG M2; Sigma-Aldrich) before collecting the beads. Beads were then washed 3 times in lysis buffer and twice in TEV buffer (10mM Tris hydrochloride, 150mM sodium chloride, 0.1% NP-40, 0.5mM EDTA, 1mM Dithiothreitol) to remove unbound proteins. Bead bound proteins were dissociated by incubation with 100U AcTEV protease (Invitrogen) in TEV buffer before collection of the eluates. The eluates were then diluted in calmodulin binding buffer (CBB; 10mM Tris-HCl, 150mM sodium chloride, 1mM magnesium acetate, 1mM Imidazole, 2mM calcium chloride, 10mM β-mercaptoethanol, 0.1% NP-40) before interacting proteins were further purified by incubation of the diluted eluates with calmodulin binding peptide (CBP) affinity resin (Agilent), pelleting and removal of the supernatant. CBP resin was then washed 3 times with CBB before incubation in calmodulin elution buffer (10mM Tris-HCl, 150mM sodium chloride, 0.02% NP-40, 1mM magnesium acetate, 1mM Imidazole, 20mM EGTA, 10mM β-mercaptoethanol) to dissociate bound proteins, which were collected in the eluates after pelleting of the CBP resin. The eluates were then snap frozen and stored at -80^oC until mass spectrometry was performed.

Samples were mixed with SDS loading buffer and run on 12% SDS PAGE gel to remove MS incompatible detergents. Samples were only run till a dye front was about 2cm below the well. Gel was stained with Coomassie blue colloidal stain and each lane cut into 4 equal pieces. Each piece was finely chopped and transferred to 0.5ml Eppendorf tube and was treated as individual MS sample. The gel pieces were distained by washing with 50% acetonitrile, 50% ammonium bicarbonate followed by wash with HPLC water. Distained samples were then treated with 100% acetonitrile, air dried and reduced with 10mM DTT in 100mM ammonium bicarbonate for 1 hour at 37^OC. The DTT solution was removed and replaced with 55mM iodoacetamide in 100mM

ammonium bicarbonate and incubated for 45 minutes at RT in darkness. Samples were then washed with 50% ammonium bicarbonate, 50% acetonitrile followed by addition of 100% acetonitrile. Acetonitrile was removed and samples dried again, before resuspending in a 1:200 solution of trypsin in 50mM ammonium bicarbonate and incubating overnight at 37°C.

LC-MS/MS analysis

Post-trypsin digestion, the supernatant was pipetted into a sample vial and loaded onto an autosampler for automated LC-MS/MS analysis.

All LC-MS/MS experiments were performed using a Dionex Ultimate 3000 RSLC nanoUPLC system and a Q Exactive Orbitrap mass spectrometer (both Thermo Fisher Scientific). Separation of peptides was performed by reverse-phase chromatography at a flow rate of 300nL/min and a Thermo Scientific reverse-phase nano Easy-spray column (Thermo Scientific PepMap C18, 2µm particle size, 100Å pore size, 75nm i.d. x 50cm length). Peptides were loaded onto a pre-column (Thermo Scientific PepMap 100 C18, 5µm particle size, 100Å pore size, 300nm i.d. x 5mm length) from the Ultimate 3000 autosampler with 0.1% formic acid for 3 minutes at a flow rate of 10nL/min. After this period, the column valve was switched to allow elution of peptides from the pre-column onto the analytical column. Solvent A was water + 0.1% formic acid and solvent B was 80% acetonitrile, 20% water + 0.1% formic acid. The linear gradient employed was 2-40% B in 30 minutes.

The LC eluent was sprayed into the mass spectrometer by means of an Easy-Spray source (ThermoFisher Scientific). All *m/z* values of eluting ions were measured in an Orbitrap mass analyzer, set at a resolution of 70000 and was scanned between *m/z* 380-1500. Data dependent scans (Top 20) were employed to automatically isolate and generate fragment ions by higher energy collisional dissociation (HCD, NCE:25%) in the HCD collision cell and measurement of the resulting fragment ions was performed in the Orbitrap analyser, set at a resolution of 17500. Singly charged ions and ions with unassigned charge states were excluded from being selected for MS/MS and a dynamic exclusion window of 20 seconds was employed.

Post-run, the data was processed using Protein Discoverer (version 2.1., Thermo Scientific). Briefly, all MS/MS data were converted to mgf files and the files were then submitted to the Mascot search algorithm (Matrix Science) and searched against the UniProt human database (UniProt_Human201701 201701, 70956 sequences; 23911858 residues) and a common contaminant sequences (115 sequences, 38274 residues). Variable modifications of oxidation (M), deamidation (NQ) and carbamidomethyl were applied. The peptide and fragment mass tolerances were set to

5ppm and 0.1 Da, respectively. A significance threshold value of $p<0.05$, false discovery rate (FDR) was set at 5% for identification of both proteins and peptides and a peptide cut-off score of 20 were also applied. Data is presented as unique peptide counts and normalised total spectra, which normalises the spectral count for each peptide in each sample to the average total spectral count across all samples.

Cloning, production, purification and structural solution of amino acid 174-442 of CRLF3

Crlf3 amino acid 174-442 was amplified from murine *in vitro* cultured MK cDNA by PCR using specific primers that include the BamHI or NotI restriction sites and Platinum High Fidelity Taq Polymerase (Invitrogen) following manufacturer's instructions. Purified PCR products were ligated into BamHI and NotI digested pGEX-6P-2 (GE Healthcare) using T4 Ligase (New England Biolabs) following manufacturer's instructions. pGEX vectors containing *Crlf3* constructs were transformed into BL21-CodonPlus™-(DE3)-RP competent *E.coli* cells (Stratagene) following manufacturer's instructions. Glutathione-*S*-transferase (GST) fusion protein from *Crlf3* constructs were produced as previously reported[55]. GST was removed from CRLF3 constructs by affinity chromatography using GSTrap FF 5mL columns and PreScission Protease (both GE Healthcare), following manufacturer's instructions. Remaining free GST was removed by ion exchange chromatography using HiTrap Q FF columns (GE Healthcare) following manufacturer's instructions. Protein was concentrated under centrifugation using VivaSpin 2 columns with 10kDa cut off (Sartorius Stedim Biotech) following manufacturer's instructions. Crystallisation was screened by the vapour diffusion method in 96-well sitting drop plates set up with a Nanodrop Screenmaker 96+8 (Innovadyne Technologies), using drops containing 200nL protein and 200nL screening solution equilibrated with 70µL screening solution. Crystals from Screen plates Structure Screen 1 and 2, PACT Premier, Stura Footprint Screen, MacroSol and JCSG-Plus (all Molecular Dimensions), Wizard 1, 2, 3 and 4 and Cryo I and II (all Emerald BioSystems) and PEG/Ion Screen and PEG/Ion 2 Screen (both Hampton Research) were used. Screen plates were kept at 20°C and imaged using a Rock Imager crystal screening system (Formulatrix). Crystals from a range of conditions were picked for X-ray diffraction and cryoprotected for data collection at 100 K by adding 25% ethylene glycol (Hampton Research) or (in the case of the native data) coating the crystal in perfluoropolyether oil. A number of crystals underwent heavy atom soaking before cryoprotection. Diffraction data were recorded at Diamond Light Source (Didcot, UK), using beamline I04 for native and Hg-SAD data and beamline I02 for long-wavelength native. Diffraction data were indexed, integrated, and scaled using Mosflm[7] and Aimless[8] via the xia2 automated data processing pipeline[9]. Long-wavelength (1.7001 Å) native data were collected to a resolution of 1.74

Å from a crystal grown in 12 % PEG 3350, 0.1M sodium acetate, but attempts to solve the structure of CRLF3 construct 3 by S-SAD phasing failed, perhaps because of significant radiation damage. The structure was determined by Hg-single anomalous diffraction (SAD) using the AutoSol-wizard[10] of the PHENIX suite[11] with a dataset collected with a wavelength of 1.006 Å from a crystal grown in 20% PEG 3350, 0.2M sodium formate, pH 7.0, soaked with 10mM thimerosal (Sigma-Aldrich) for 16 hours. Native data were collected with a wavelength of 0.9795 Å from a crystal grown in 25% PEG 3350, 0.2 M ammonium acetate, 0.1 M Bis-Tris, pH 7.5. The structure was refined to a resolution of 1.61 Å using Phenix.refine[11] and by manual building in Coot[12]. The final model has good statistics, with 96% of the chain in the Ramachandran favoured region and no Ramachandran outliers. Other crystallographic statistics are presented in Supplemental Table 3.

Supplemental Tables (Tables 2, 4, 5 and 7 are found in an additional excel file)

Supplemental Table 1: CRLF3 deficient mice (*Crlf3*$^{-/-}$) have an isolated reduction in platelet count

	Male		Female	
	WT	*Crlf3*$^{-/-}$	WT	*Crlf3*$^{-/-}$
**Young**				
Plt (10^3/μL)	1424 (251)	875 (170)***	1117 (261)	686 (105)***
MPV (fL)	5.01 (0.38)	5.19 (0.37)	4.96 (0.09)	5.08 (0.15)
Hgb (g/dL)	14.51 (1.16)	13.51 (1.42)	15.72 (2.33)	15.02 (1.58)
RBC (10^6/μL)	10.32 (1.38)	9.46 (1.48)	10.21 (1.70)	9.68 (1.23)
WBC (10^3/μL)	11.15 (2.64)	13.56 (5.71)	7.80 (1.44)	7.40 (3.46)
Age (weeks)	15.34 (2.93)	15.55 (3.38)	16.83 (2.90)	14.83 (2.39)
n	10	7	5	5
**Middle aged**				
Plt (10^3/μL)	1485 (205)	1079 (203)***	1035 (133)	771 (134)***
MPV (fL)	4.78 (0.23)	5.02 (0.22)	4.96 (0.09)	5.13 (0.14)
Hgb (g/dL)	13.39 (1.88)	13.08 (1.23)	13.42 (0.97)	13.83 (1.03)
RBC (10^6/μL)	9.92 (1.56)	9.17 (0.80)	8.71 (0.75)	9.04 (0.60)
WBC (10^3/μL)	9.71 (2.70)	8.38 (1.06)	6.86 (1.34)	5.70 (1.38)
Age (weeks)	31.17 (5.27)	27.31 (4.15)	27.14 (4.30)	25.86 (4.69)
n	10	5	5	6
**Old**				
Plt (10^3/μL)	1514 (444)	1132 (384)***	1324 (242)	772 (162)***
MPV (fL)	5.30 (0.42)	5.21 (0.60)	5.50 (0.64)	5.42 (0.39)
Hgb (g/dL)	13.86 (0.75)	12.43 (1.44)	13.47 (1.73)	13.24 (1.33)
RBC (10^6/μL)	10.34 (1.03)	9.02 (1.55	9.57 (1.65)	8.95 (1.36)
WBC (10^3/μL)	10.53 (3.18)	12.30 (3.36)	6.18 (0.95)	7.96 (3.47)
Age (weeks)	65.39 (13.21)	64.94 (14.43)	69.01 (14.56)	65.55 (15.40)
n	12	23	11	14

Full blood counts determined by an automated haemocytometer on EDTA anticoagulated venous blood taken from male and female young (12-20 weeks), middle aged (21-40 weeks) and old (>48 weeks) mice. Data represents mean (SD). Unpaired 2-tailed Student's *t* test with correction for multiple comparisons using the Holm-Sidak method. *** denotes p<0.005. Plt - platelet count; MPV - mean platelet volume; HgB - haemoglobin concentration; RBC - red blood cell count; WBC - white blood cell count.

Supplemental Table 3: Data collection and refinement statistics

	Hg-SAD	Native 1	S-SAD
**Data collection**			
Wavelength (Å)	1.006	0.9795	1.7001
Space group	C2	C2	C2
Cell dimensions			
a, *b*, *c* (Å)	87.25, 40.94, 76.29	88.03, 40.38, 76.53	87.80, 40.60, 76.66
α, β, γ (°)	90, 98.74, 90	90, 99.94, 90	90, 99.80, 90
Resolution (Å)	27.3-1.97 (2.02-1.97)*	37.7-1.61 (1.65-1.61)	32.1-1.74 (1.79-1.74)
R_{meas}	0.095 (0.861)	0.066 (1.100)	0.067 (0.930)
$I / \sigma I$	16.6 (2.1)	13.3 (1.4)	20.0 (2.1)
Completeness (%)	99.7 (96.0)	96.7 (94.9)	88.4 (49.2)
Redundancy	6.7 (6.7)	3.4 (3.6)	9.3 (6.4)
$CC_{1/2}$	0.999 (0.829)	0.999 (0.504)	0.999 (0.492)
CC_{anom}	0.689 (0.039)	—	0.323 (0.040)
**Refinement**			
Resolution (Å)	27.3-1.97	37.7-1.61	32.1-1.74
No. refl. work / free	19150 / 907	31712 / 1613	23106 / 1204
R_{work} / R_{free}	0.186 / 0.234	0.177 / 0.201	0.177 / 0.200
No. atoms			
Protein	1951	2000	2011
Ligand/ion	11	0	0
Water	93	108	166
B-factors			
Protein (Å^2)	39.5	36.8	38.8
Water (Å^2)	41.8	32.1	42.3
R.m.s. deviations			
Bond lengths (Å)	0.012	0.009	0.011
Bond angles (°)	1.134	1.066	1.001
PDB ID	6RPY	6RPX	6RPZ

*Values in parentheses are for highest-resolution shell.

Supplementary Table 6: Full blood counts of *Crlf3*$^{-/-}$ x JAK2V617F ET animals

	Plt ($10^3/\mu L$)	MPV (fL)	Hgb (g/dL)	RBC ($10^6/\mu L$)	WBC ($10^3/\mu L$)	Age (weeks)	*n*
**Young**							
1 - WT control	875 (306)	5.62 (0.26)	14.52 (0.75)	10.52 (0.54)	10.37 (4.24)	10.22 (3.46)	6
2 - Crlf3$^{-/-}$	686 (222)	5.71 (0.32)	13.63 (1.48)	9.98 (1.22)	8.90 (2.93)	9.49 (3.38)	7
3 - JAK2V617F ET	1489 (255)	5.64 (0.36)	14.79 (0.94)	10.68 (0.63)	10.16 (2.27)	12.53 (4.79)	7
4 - Crlf3$^{-/-}$ JAK2V617F	879 (229)	5.83 (0.53)	14.78 (1.54)	10.93 (1.27)	11.42 (2.35)	12.31 (4.24)	6
Statistical testing							
1 v 2	***	ns	ns	ns	ns	ns	
1 v 3	***	ns	ns	ns	ns	ns	
1 v 4	ns	ns	ns	ns	ns	ns	
2 v 3	***	ns	ns	ns	ns	ns	
2 v 4	***	ns	ns	ns	ns	ns	
3 v 4	***	ns	ns	ns	ns	ns	
**Old**							
1 - WT control	1030 (426)	5.46 (0.52)	13.90 (1.19)	10.24 (0.41)	12.42 (4.13)	57.86 (7.77)	5
2 - Crlf3$^{-/-}$	712 (227)	5.31 (0.59)	13.54 (1.77)	9.55 (1.42)	8.00 (2.06)	65.74 (8.17)	7
3 - JAK2V617F ET	1446 (276)	5.30 (0.47)	13.58 (1.33)	10.23 (1.05)	9.27 (3.01)	68.06 (11.05)	9
4 - Crlf3$^{-/-}$ JAK2V617F	890 (137)	5.12 (0.34)	14.97 (2.23)	11.13 (1.66)	8.72 (0.68)	71.95 (16.10)	6
Statistical testing							
1 v 2	***	ns	ns	ns	ns	ns	
1 v 3	***	ns	ns	ns	ns	ns	
1 v 4	ns	ns	ns	ns	ns	ns	
2 v 3	***	ns	ns	ns	ns	ns	
2 v 4	*	ns	ns	ns	ns	ns	
3 v 4	***	ns	ns	ns	ns	ns	

Full blood counts determined using an automated haemocytometer on EDTA anticoagulated venous blood withdrawn from young (≤20-week-old) and old (≥48-week-old) female WT control, Crlf3$^{-/-}$, JAK2V617F ET and Crlf3$^{-/-}$ JAK2V617F mice. Data represents mean (SD). Two-way ANOVA with correction for multiple comparisons using the Holm-Sidak method. *, *** and ns denote $p<0.05$, $p<0.005$ and non-significant, respectively. Plt - platelet count; MPV - mean platelet volume; HgB - haemoglobin concentration; RBC - red blood cell count; WBC - white blood cell count.

Supplemental Figures

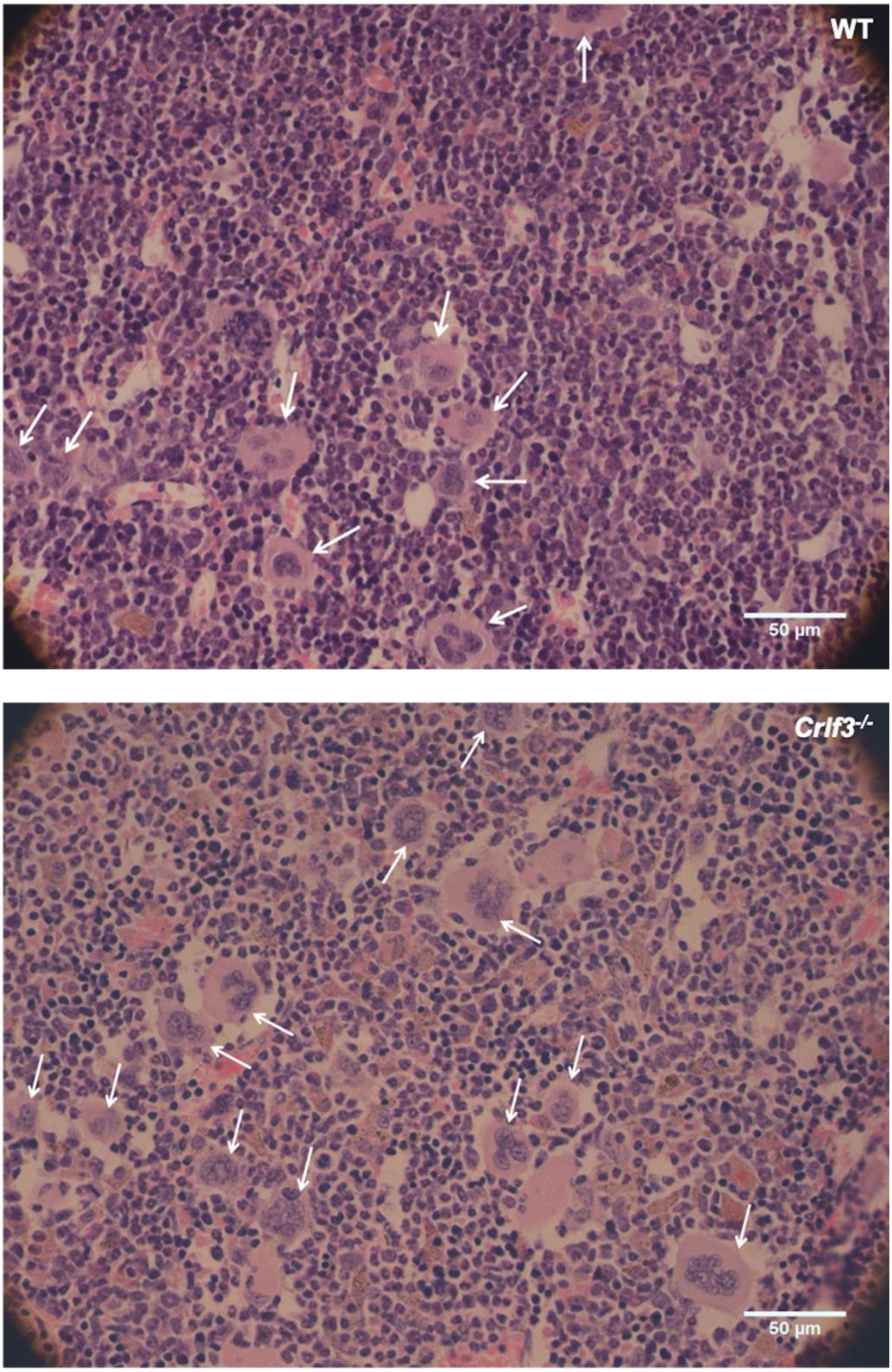

Supplemental Figure 1: Quantification of bone marrow residing MKs

Control (WT) and *Crlf3*$^{-/-}$ tibia were fixed, sectioned, and stained with Haematoxylin and eosin (H&E). MKs were quantified after imaging by light microscopy. Images are representative of each genotype and arrows point to MKs. Scale bars are 50µm.

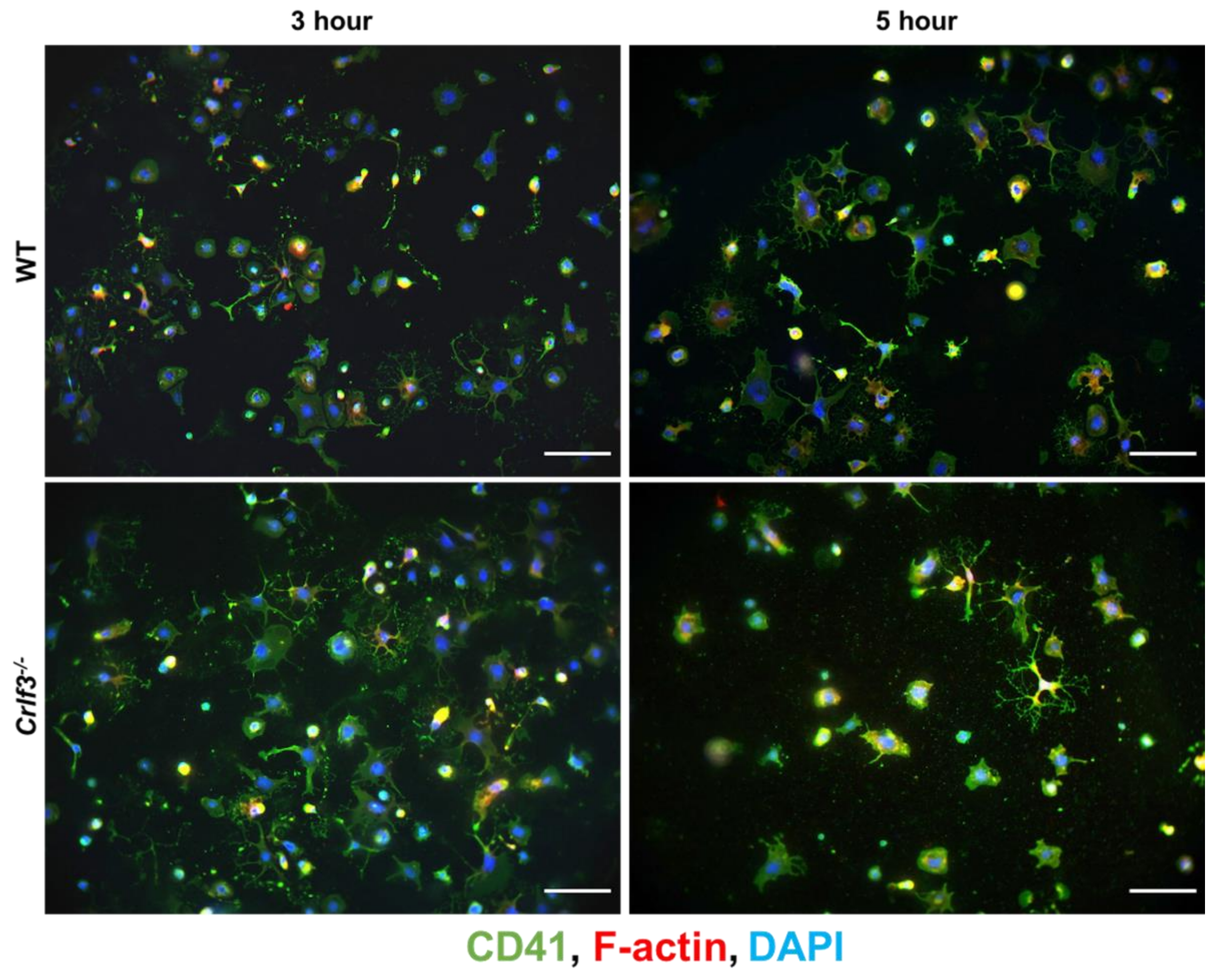

Supplemental Figure 2: CRLF3 deficiency increases speed of proplatelet formation

In vitro cultured MKs were seeded onto fibrinogen coated coverslips and incubated at 37ºC for 3 or 5 hours to induce proplatelet formation. Samples were fixed, stained with CD41 (green), F-actin (red) and DAPI (blue), and imaged by confocal microscopy. Images are representative of each genotype at each time point. Scale bars are 50µm.

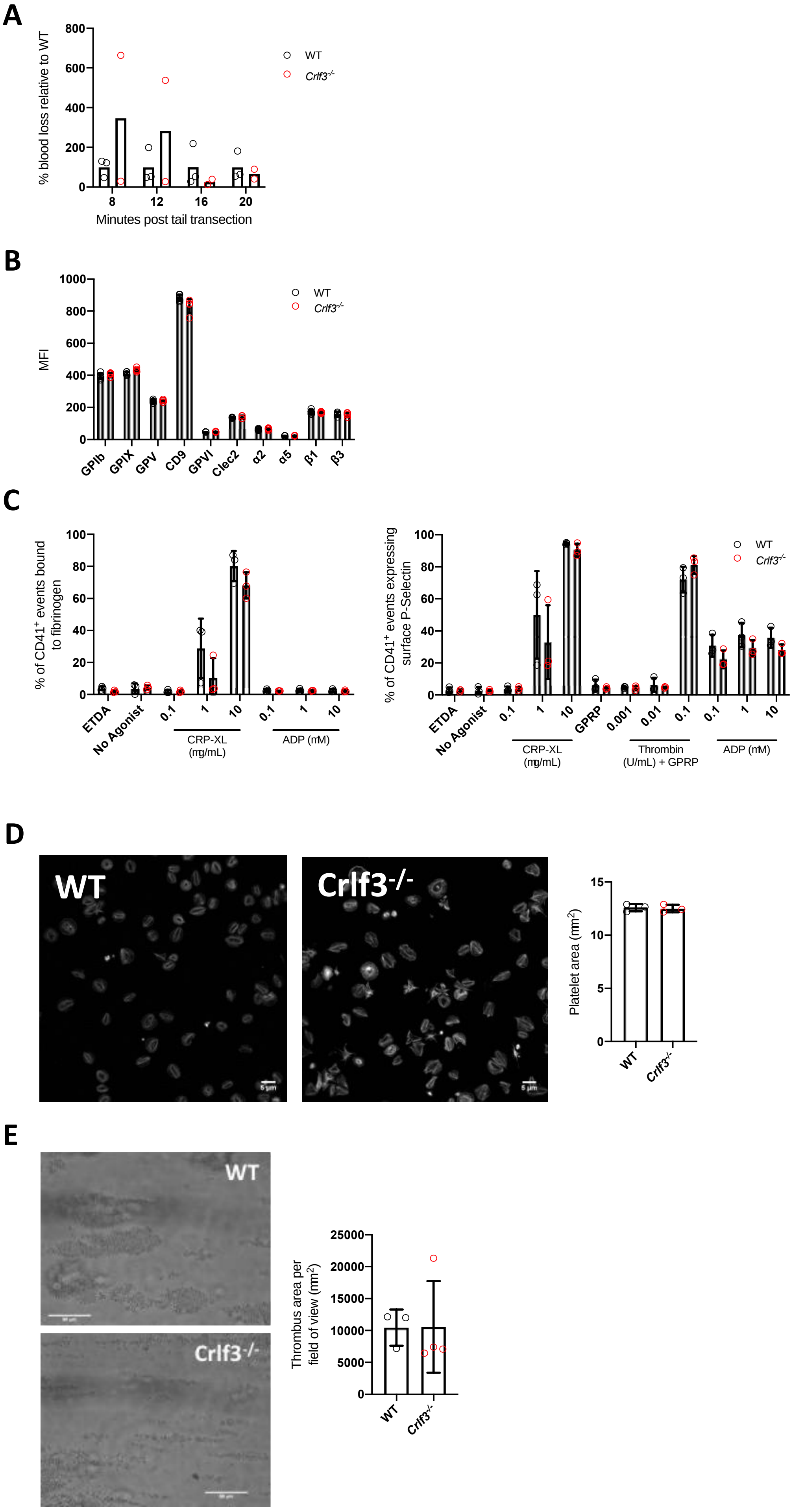

A
% blood loss relative to WT
Minutes post tail transection
WT
Crlf3-/-
B
MFI
GPIb
GPIX
GPV
CD9
GPVI
Clec2
α2
α5
β1
β3
C
% of CD41+ events bound to fibrinogen
% of CD41+ events expressing surface P-Selectin
ETDA
No Agonist
CRP-XL (mg/mL)
ADP (mM)
GPRP
Thrombin (U/mL) + GPRP
D
WT
Crlf3-/-
Platelet area (mm2)
E
Thrombus area per field of view (mm2)

Supplemental Figure 3: CRLF3 deficiency does not affect platelet function

(**A**) Control (WT, black) and *Crlf3*$^{-/-}$ (red) blood loss after removal of tail tip. (**B**) Control (WT, black) and *Crlf3*$^{-/-}$ (red) whole blood incubated with antibodies against the membrane proteins GPIb, GPIX, GPV, CD9, GPVI, Clec2, α2, α5, β1 and β3 and assessed by flow cytometry, gating on platelet sized events (*n*=5). (**C**) Control (WT, black) and *Crlf3*$^{-/-}$ (red) whole blood incubated with specific antibodies against CD41, P-Selectin and fibrinogen with or without addition of agonists. Percentage of CD41^{+} events bound to fibrinogen (left panel) or expressing surface P-selectin (right panel) was determined by flow cytometry (*n*=3). (**D**) Control (WT, black) and *Crlf3*$^{-/-}$ (red) washed platelets seeded onto fibrinogen coated coverslips and incubated for 30 mins at 37°C, fixed, stained for F-actin and imaged by fluorescence microscopy. Platelet area determined by thresholding images on F-actin staining using ImageJ (*n*=3). (**E**) Heparinised whole blood flowed at arterial shear rate (1,000 s^{-1}) over a surface coated with fibrillar collagen and imaged by light microscopy. Thrombus area for control (WT, black) and *Crlf3*$^{-/-}$ (red) mice determined by manually measuring the size of each thrombi using ImageJ (*n*=3 WT/4 *Crlf3*$^{-/-}$). Data represents mean ± SD. Unpaired 2-tailed Student's *t* test (**D** and **E**) with correction for multiple comparisons using the Holm-Sidak method (**B** and **C**). MFI = mean fluorescent intensity.

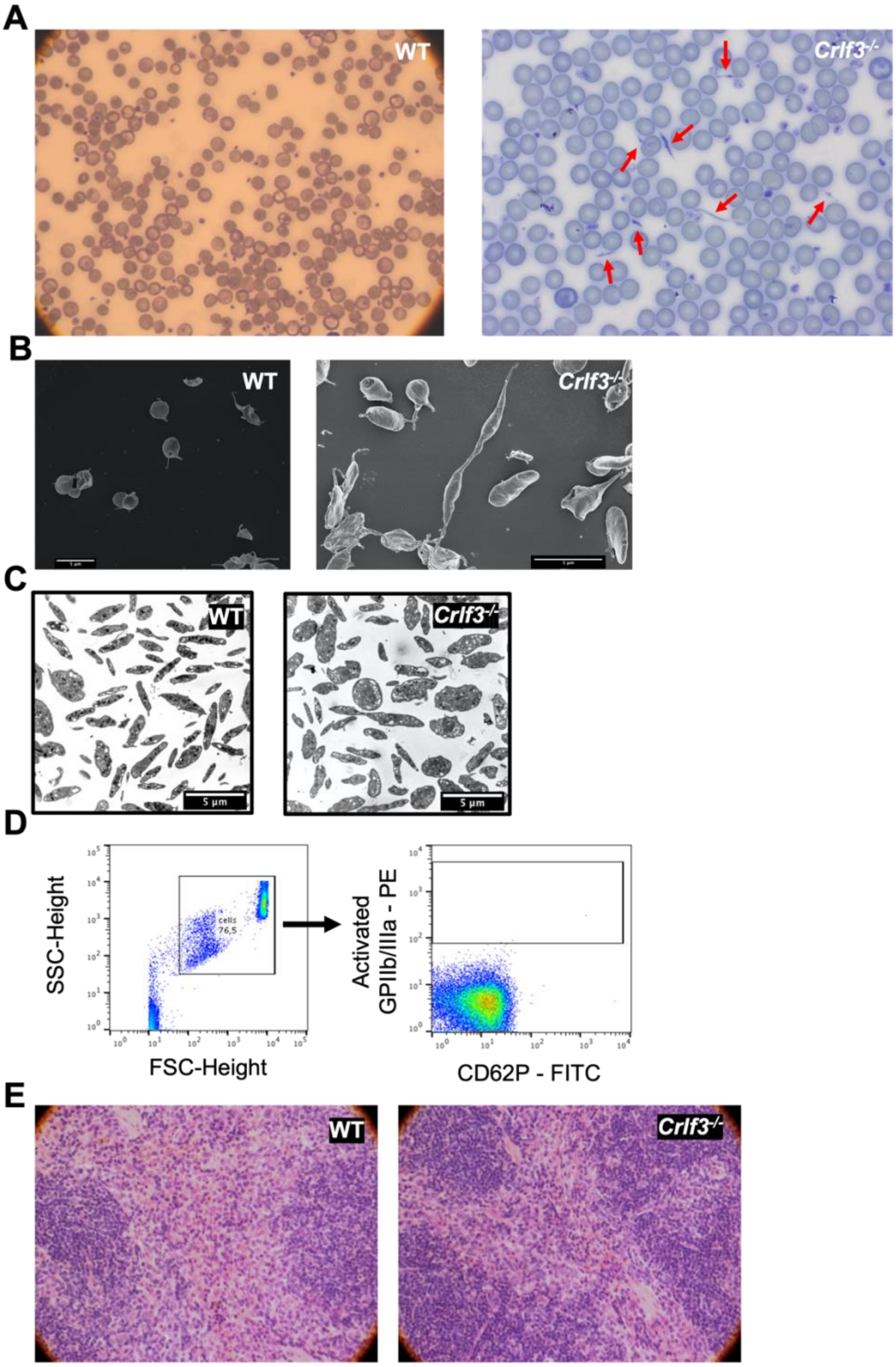

Supplemental Figure 4: CRLF3 deficiency causes ineffective thrombopoiesis

(**A**) Blood smears from control (WT) and *Crlf3*$^{-/-}$ whole blood taken at 63x and 100x magnification under light microscopy, respectively. Red arrows point to elongated spindle form preplatelet structures. (**B**) Control (WT) and *Crlf3*$^{-/-}$ washed platelets were fixed and prepared for scanning and (**C**) transmission electron microscopy. Scale bars are 5µm. (**D**) To rule out the possibility that preplatelets are platelet microaggregates, heparinised venous blood was incubated with specific antibodies against activated GPIIb/IIIa and CD62P and assessed by flow cytometry. (**E**) Hematoxylin and eosin-stained spleen sections from control (WT) and *Crlf3*$^{-/-}$ mice imaged at 20x magnification under light microscopy.

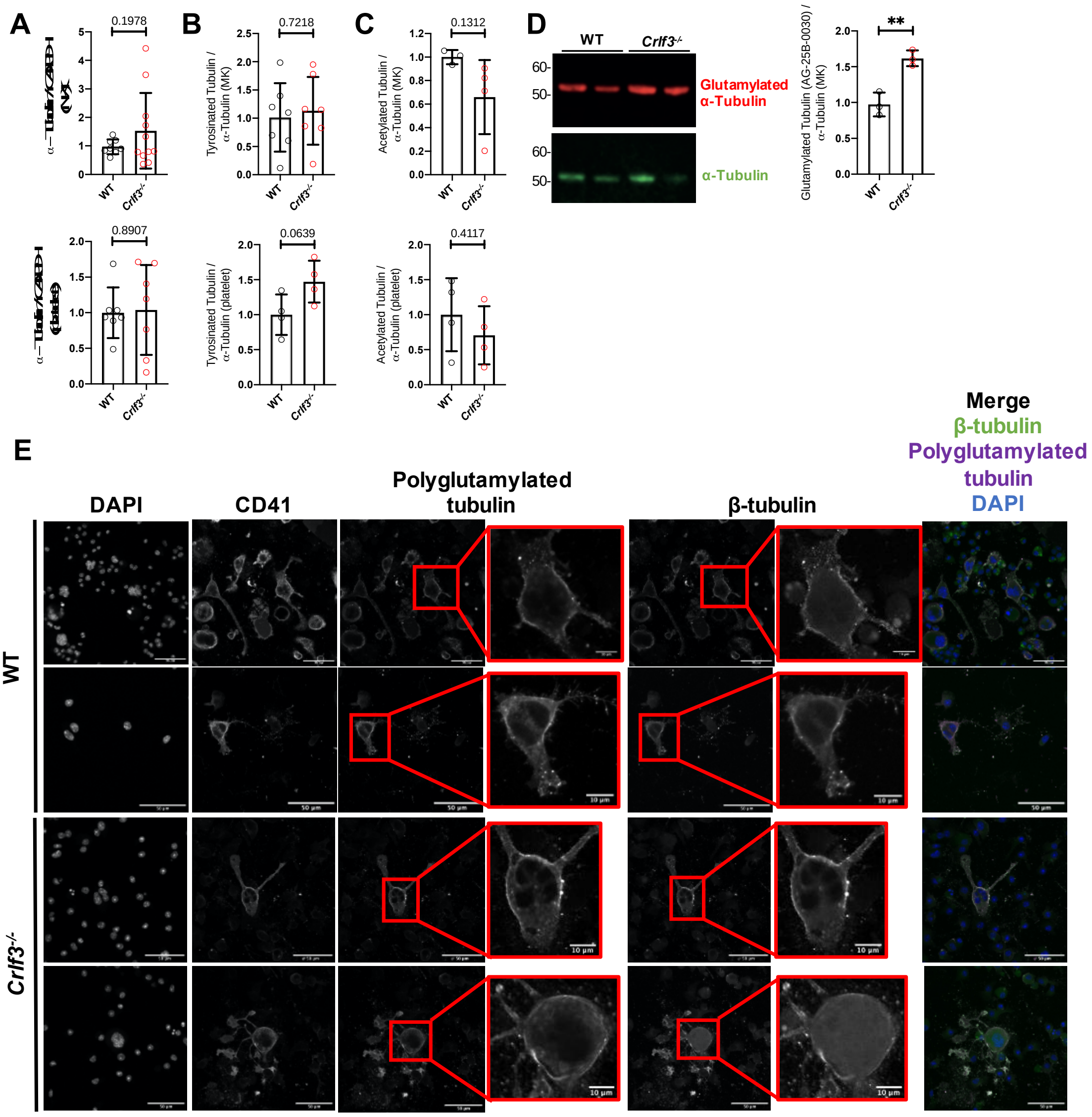

Supplemental Figure 5: Tubulin modifications in MKs and platelets

(**A**) Densitometry quantification of α-Tubulin relative to GAPDH (*n*=7 platelet; *n*=8 WT/11 *Crlf3*$^{-/-}$ MK), (**B**) tyrosine α-Tubulin relative to α-Tubulin (*n*=4 platelet; *n*=7 MK), (**C**) acetylated α-Tubulin relative to α-Tubulin (*n*=4 platelet; *n*=3 WT/4 *Crlf3*$^{-/-}$ MK) and (**D**) polyglutamylated tubulin (AG-25B-0030, AdipoGen) relative to α-Tubulin (n=3 MK) from western blots of *in vitro* cultured MK (top panels) and platelet (bottom panels) lysates from control (WT, back) and *Crlf3*$^{-/-}$ (red) animals probed with specific antibodies. (**E**) *In vitro* cultured MKs were seeded onto fibrinogen coated coverslips and incubated at 37°C for 5 hours to induce proplatelet formation. Samples were fixed, stained for CD41, polyglutamylated α-Tubulin (AG-25B-0030; magenta), β-tubulin (green) and DAPI (blue) imaged by fluorescence microscopy. Images are representative for *Crlf3*$^{-/-}$ and control (WT) proplatelet forming MKs. Scale bars are 50µm and 10µm in the boxed zoomed images. Data represents mean or mean± SD. Unpaired 2-tailed Student's or Welch's (A - MK only) t test.

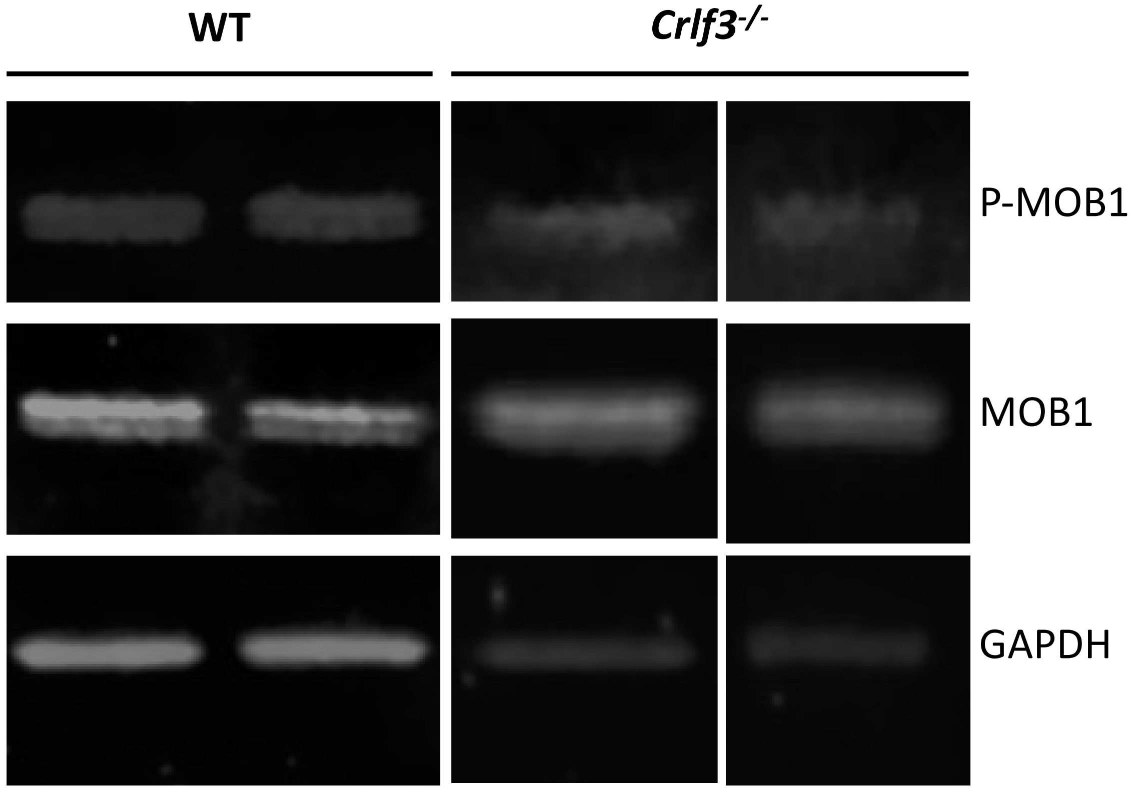

Supplemental Figure 6: Mob1 phosphorylation in control and *Crlf3$^{-/-}$* MK

Western blot of WT (control) and *Crlf3$^{-/-}$* *in vitro* cultured MKs probed with specific antibodies against phosphorylated MOB1 (P-MOB1), MOB1 and GAPDH.

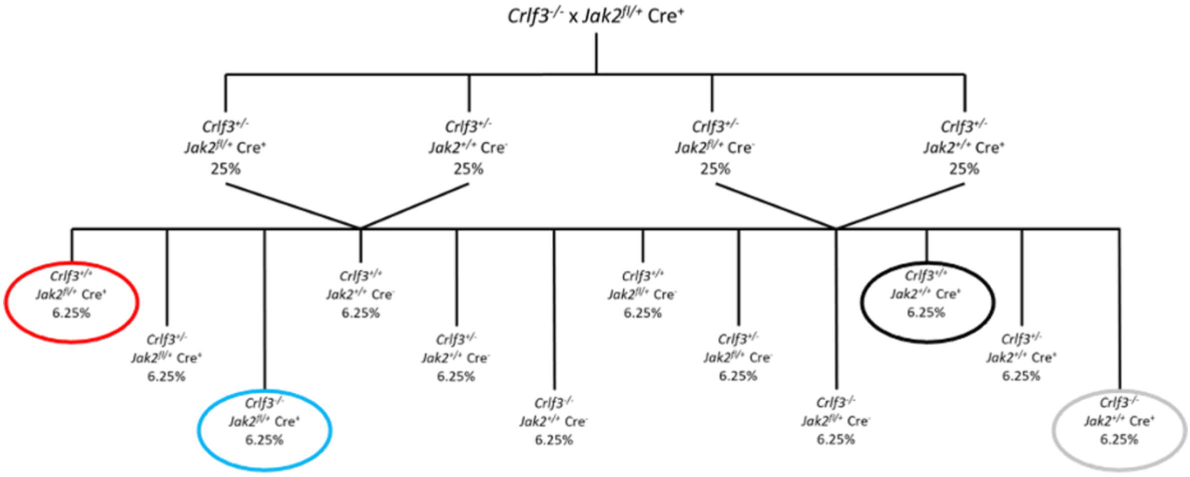

Supplemental Figure 7: Breeding strategy to study effect of *Crlf3* ablation on essential thrombocythaemia

Crlf3$^{-/-}$ mice were crossbred with JAK2V617F Essential Thrombocythaemia (ET) mice[13] generating *Crlf3* heterozygous intermediates, which were cross bred again to generate lines of interest. Genotypes of interest are circled (black = WT control; grey = *Crlf3*$^{-/-}$; red = *JAK2V617F* ET and green = *Crlf3*$^{-/-}$ *JAK2V617F*).

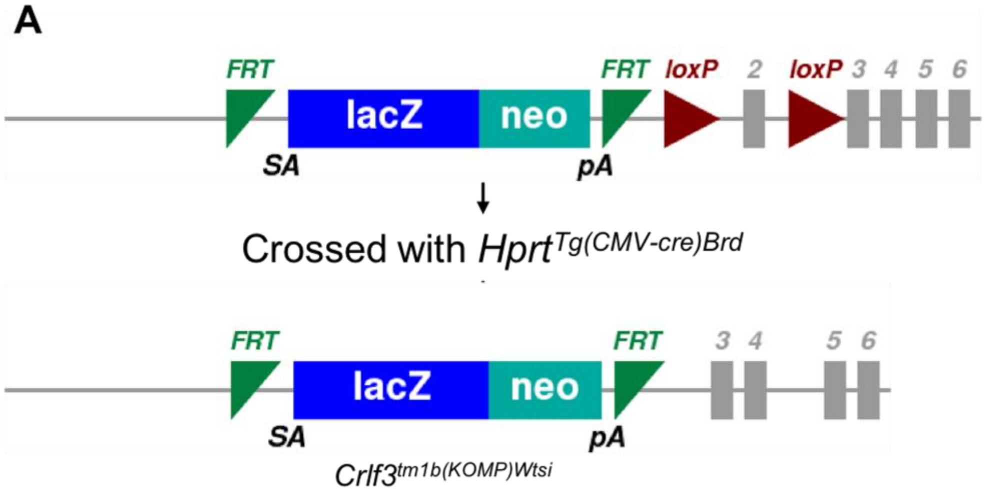

B

Primer	Sequence (5'>3')
Crlf3_F	TGGATACATTGATTAGCTTGACAGA
Crlf3_R	AGGGGATGAGAGGGTCTTCAG
Cassette_R	TCGTGGTATCGTTATGCGCC

PCR primer pairs and expected sizes			
**Allele**	**Forward primer**	**Reverse Primer**	**Expected PCR product size (bp)**
Wildtype	Crlf3_F	Crlf3_R	281
Mutant	Crlf3_F	Cassette_R	159

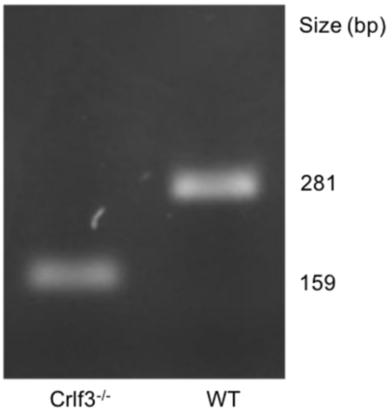

Supplemental Figure 7: Generation of *Crlf3*$^{tm1b(KOMP)Wtsi}$ (*Crlf3*$^{-/-}$) mice (**A**) The allele structure for *Crlf3*. Exon 2 was excised by cre-mediated recombination. (**B**) Primers used for mouse genotyping, expected band size and representative image of a genotyping assay.

Videos

Video 1 and 2: Proplatelet formation is not impaired in *Crlf3*$^{-/-}$ mice

Anesthetised Control (WT; **Video 1**) and *Crlf3*$^{-/-}$ (**Video 2**) mice injected with tetramethylrhodamine dextran and anti-CD105 (both red) to label bone vasculature and anti-GPIX (green) to label platelets and MKs. Fronto-parietal skull exposed, and images acquired every 5 seconds by fluorescence microscopy and videos created using ImageJ. Scale bars are 50µm.

References

1. Hobbs CM, Manning H, Bennett C, et al. JAK2V617F leads to intrinsic changes in platelet formation and reactivity in a knock-in mouse model of essential thrombocythemia. *Blood*. 2013;122(23):3787-3797.
2. Mayer L, Jasztal M, Pardo M, et al. Nbeal2 interacts with Dock7, Sec16a, and Vac14. *Blood*. 2018;131(9):1000-1011.
3. Evans AL, Dalby A, Foster HR, et al. Transfer to the clinic: refining forward programming of hPSCs to megakaryocytes for platelet production in bioreactors. *Blood Adv*. 2021;5(7):1977-1990.
4. Hodgkins A, Farne A, Perera S, et al. WGE: a CRISPR database for genome engineering. *Bioinformatics*. 2015;31(18):3078-3080.
5. Ran FA, Hsu PD, Wright J, Agarwala V, Scott DA, Zhang F. Genome engineering using the CRISPR-Cas9 system. *Nat Protoc*. 2013;8(11):2281-2308.
6. Pardo M, Lang B, Yu L, et al. An expanded Oct4 interaction network: implications for stem cell biology, development, and disease. *Cell Stem Cell*. 2010;6(4):382-395.
7. Leslie AGW, Powell HR. Processing diffraction data with mosflm. In: Read RJ, Sussman JL, eds. Evolving Methods for Macromolecular Crystallography: The Structural Path to the Understanding of the Mechanismof Action of CBRN Agents. Dordrecht: Springer Netherlands; 2007:41-51.
8. Evans PR. An introduction to data reduction: space-group determination, scaling and intensity statistics. *Acta Crystallogr D Biol Crystallogr*. 2011;67(Pt 4):282-292.
9. Winter G. xia2: an expert system for macromolecular crystallography data reduction. *Journal of Applied Crystallography*. 2010;43(1):186-190.
10. Terwilliger TC, Adams PD, Read RJ, et al. Decision-making in structure solution using Bayesian estimates of map quality: the PHENIX AutoSol wizard. *Acta Crystallogr D Biol Crystallogr*. 2009;65(Pt 6):582-601.
11. Adams PD, Afonine PV, Bunkoczi G, et al. PHENIX: a comprehensive Python-based system for macromolecular structure solution. *Acta Crystallogr D Biol Crystallogr*. 2010;66(Pt 2):213-221.
12. Emsley P, Lohkamp B, Scott WG, Cowtan K. Features and development of Coot. *Acta Crystallogr D Biol Crystallogr*. 2010;66(Pt 4):486-501.
13. Li J, Spensberger D, Ahn JS, et al. JAK2 V617F impairs hematopoietic stem cell function in a conditional knock-in mouse model of JAK2 V617F-positive essential thrombocythemia. *Blood*. 2010;116(9):1528-1538.
